# Odor source localization of multi-robots with swarm intelligence algorithms: A review

**DOI:** 10.3389/fnbot.2022.949888

**Published:** 2022-11-30

**Authors:** Junhan Wang, Yuezhang Lin, Ruirui Liu, Jun Fu

**Affiliations:** Artificial Intelligence of Things and Robotics Laboratory, School of Computer Science and Information Engineering, Zhejiang Gongshang University, Hangzhou, China

**Keywords:** odor source localization, swarm intelligence algorithm, multi-robot system, particle swarm optimization, mobile robot, nature-inspired computation

## Abstract

The use of robot swarms for odor source localization (OSL) can better adapt to the reality of unstable turbulence and find chemical contamination or hazard sources faster. Inspired by the collective behavior in nature, swarm intelligence (SI) is recognized as an appropriate algorithm framework for multi-robot system due to its parallelism, scalability and robustness. Applications of SI-based multi-robots for OSL problems have attracted great interest over the last two decades. In this review, we firstly summarize the trending issues in general robot OSL field through comparing some basic counterpart concepts, and then provide a detailed survey of various representative SI algorithms in multi-robot system for odor source localization. The research field originates from the first introduction of the standard particle swarm optimization (PSO) and flourishes in applying ever-increasing quantity of its variants as modified PSOs and hybrid PSOs. Moreover, other nature-inspired SI algorithms have also demonstrated the diversity and exploration of this field. The computer simulations and real-world applications reported in the literatures show that those algorithms could well solve the main problems of odor source localization but still retain the potential for further development. Lastly, we provide an outlook on possible future research directions.

## Introduction

### What is robot OSL?

In nature, finding, locating, and recognizing odor information is a fundamental skill for organisms. For example, foraging ants can use residues of pheromones to establish the shortest route back from a food source (Hölldobler et al., 1990), and male moths discover and find mates by tracking the odor released by female moths (Charlton and Cardé, 1990). Inspired by the biological phenomena, since the 1990's, some scholars have started to study how to use mobile robots equipped with chemical sensors to “actively” sniff out odor for various tasks, such as odor map construction, odor source localization, and odor source classification. This type of research can be referred to as active olfaction in a broad sense. Odor source localization (OSL) is a process in which single or multiple mobile robots use sensors to “proactively” discover chemical plumes in the environment, track them, and identify the source of the odor.

**Figure 1:**
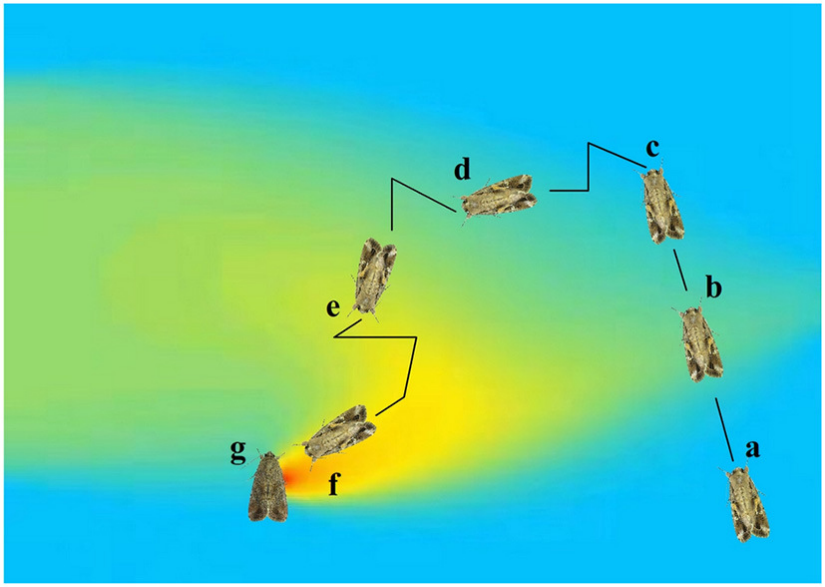
A male moth (*Spodoptera frugiperda*) **(a)** locates its mate by detecting and tracing the sex pheromone emitted from the female **(g)**, experiencing four stages: Plume finding **(b)**, plume reacquiring **(d)** after detaching from the odor coverage **(c)**, plume traversal **(e)**, and source declaration **(f)**.

The gas molecules released by the odor source are blown away by the wind and will flutter in the air like a feather to form a trajectory, which is called a plume (Murlis et al., 1992). Typically, locating odor source can be divided into three subtasks (Hayes et al., 2002): plume finding, plume traversal, and source declaration. Plume finding is a process of initial contact with the odor. Due to the stochasticity of plumes, plume finding mainly uses a random search strategy. Plume traversal is a process of making robots follow the plume to the odor source. It requires robots to have more “professional” behavior. It should move toward the odor source, and simultaneously, it cannot detach from the coverage of the odor plume. Odor source declaration refers to the robot's confirmation that the current location is the odor source rather than a local optimum. This process does not necessarily use odor information, as typical odor sources can also be determined by other means (such as vision) at short distances. Additionally, a plume reacquiring stage (Li et al., 2006) was proposed to be activated when the robots appear to lose contact with the odor for a given period during tracking plume. Figure 1 shows a male moth (*Spodoptera frugiperda*) locates its mate by detecting and tracing the sex pheromone emitted from the female, experiencing four stages.

In the early years, active olfaction for odor source localization by mobile robots did not perform as well as the fixed sensor approach. An essential reason is that under general indoor ventilation conditions, odor diffusion is influenced by turbulence, making the distribution of plumes show complex characteristics such as time-varying, interval, and multi-extremum. However, mobile robots only used chemotaxis to collect concentration information for plume tracking, making them easily trapped in the maximum local odor concentration or too long to converge, leading to failure. Over the last two decades, many studies have been conducted to make the robot avoid local optima and improve the search performance of algorithms in turbulent plumes.

Currently, there are four mainstream methods for locating odor sources (Jing et al., 2021), viz., reactive methods, heuristic search methods, probabilistic inference methods, and learning methods. The heuristic search method treats odor source localization as a functional optimization problem, that is, finding the optimal odor concentration in a particular region. Therefore, the OSL problem can be transformed into an optimal optimization problem. Among heuristic search methods, the swarm intelligence (SI) algorithms have been considered as a promising approach for the following advantages:

- Swarm intelligence algorithms mainly complete the work of complex tasks through self-organization by a population of individuals with simple behavior. The simple search behavior of a single robot is the continuation of a reactive robot based on odor concentration.
- Swarm intelligence algorithms can coordinate multiple robots to search for odor sources and effectively solve the defects of a single robot in the process of odor source localization, such as poor robustness and quickly falling into a locally optimal solution.
- In contrast to probabilistic inference methods and learning methods, the swarm intelligence algorithm mainly achieves the optimal position of odor concentration by multiple iterations of multi-robot individuals. It does not require the continuity and derivability of odor concentration to fit with the plume concentration's discontinuous and multi-local optimal characteristics in a natural turbulent environment.

Therefore, since 2006, researchers have begun to apply swarm intelligence algorithms to odor source localization in mobile robots. This paper gives an overview of odor source localization, focusing on swarm intelligence algorithms applied to this area.

### Why this review?

So far, several investigations report on robot odor source localization have been published. Lilienthal et al. (2006) sorted out the literatures and published a review. However, the review was published more than a decade ago. The experiments mentioned were carried out in the indoor scenario, mainly considering the two-dimensional search space and classifying the applicable environmental conditions. The experimental conditions considered are still significantly different from those expected in most typical applications. It shows that the challenge in the future is to evaluate how to effectively combine different algorithms and test the corresponding implementation in a natural environment. Kowadlo and Russell (2008) pointed out that 3D localization will be further developed in the future, and more consideration will be given to obstacles and unstructured environments. Ishida et al. (2012) reviewed three subtasks of robotic odor localization. They argued that robotic OSL research has moved from the simulation and experimental stage to a more practical application-oriented stage, which is instructive for subsequent research. Chen and Huang (2019) used a new classification approach in their review: using algorithmic principles at the top level of the classification hierarchy, such as how to handle input signals (such as chemotaxis and anemotaxis), classified existing OSL algorithms into four categories, and noted that gradient-based algorithms currently lack attention and that probability and map-based algorithms are more attractive for research. In a recent review, Jing et al. (2021) pointed out that the classification method of the former may lead to some overlap between different categories and proposed a stricter classification method based on the principle of method according to the literatures published in recent years, and also emphasized some aspects not discussed in previous studies: *in-situ* sensing and simulator development for odor source direction and distance prediction. Meanwhile, they pointed out that the algorithm extensions to 3D environments, multiple robots, and their mixing situation have progressed in the past 10 years. Francis et al. (2022) recently published a review to overview the research on gas distribution mapping and source localization for both controlled and uncontrolled environments with robots, focusing on probabilistic algorithms developed for both single robot and multi-robot applications.

In recent years, more and more researchers have started to solve the OSL problem with swarm intelligence, and many significant research results have been achieved. However, to the best of our knowledge, review literature related to this has not been seen reported yet, so in this paper, we review multi-robot algorithms applied to odor source localization from the perspective of swarm intelligence. We review and discuss the main issues of odor source localization in turn in the last decade. The application of particle swarm optimization (PSO) algorithm and its improved algorithms are emphasized. Hybrid particle swarm algorithms combined with other independent algorithms will also be discussed. Various bio-inspired meta-heuristic optimization methods, which were not highlighted in the past reviews and are hot research trends in recent years, will be discussed separately.

### How to categorize algorithms?

**Figure 2:**
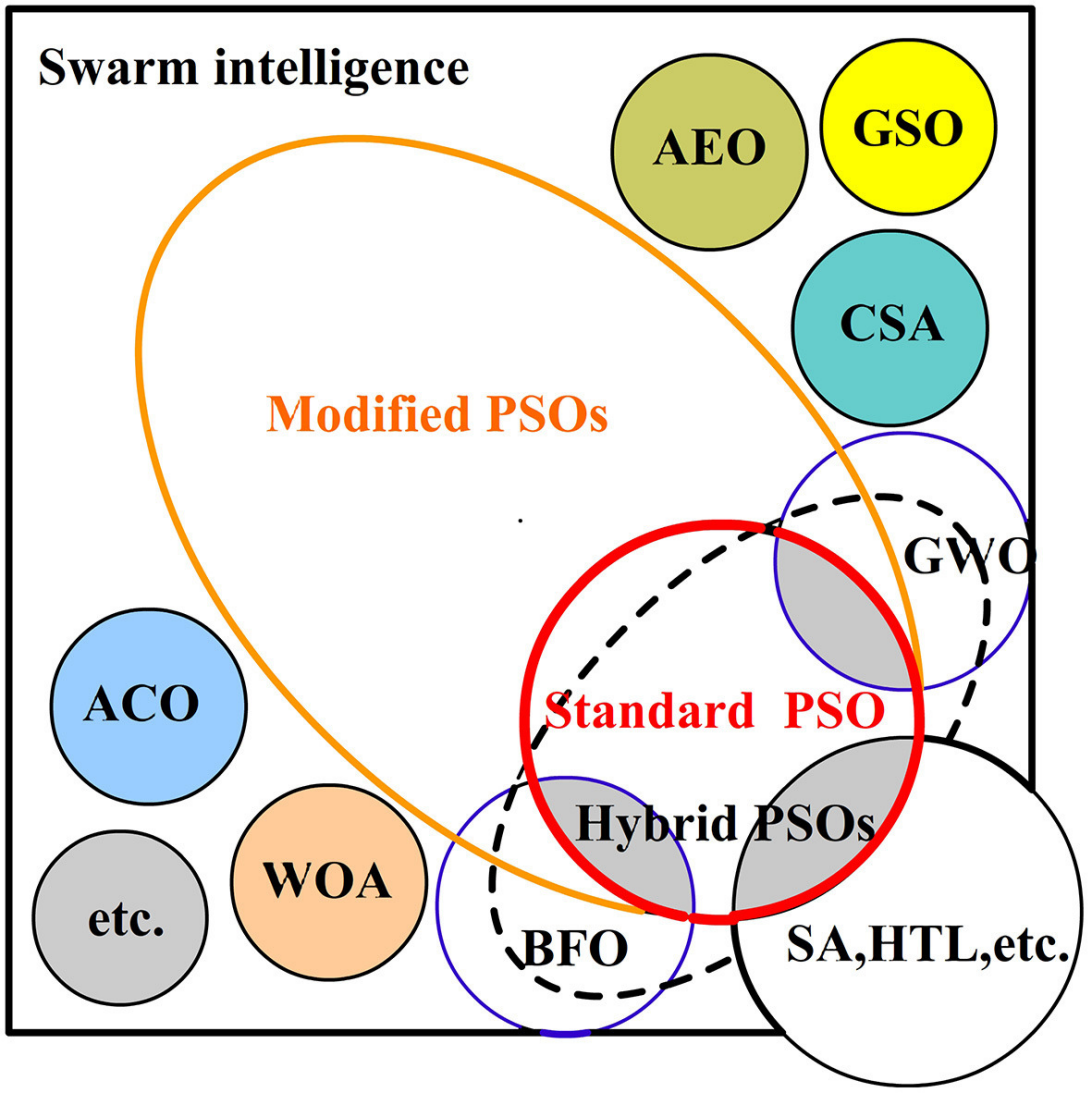
The diagram of existing swarm intelligence algorithms applied to multi-robot system for odor source localization.

The existing swarm intelligence algorithms mainly applied to multi-robot system for odor source localization (Figure 2) are classified as follows according to the algorithm principle:

*The standard PSO algorithm* for OSL heavily relies on chemotaxis. Its inherent algorithm principle allows it to coordinate multiple robots, using current information, individual history information, and global history information to localize the target.
*The modified PSO algorithms* realize the optimization of the standard PSO to adjust particle position in the next iteration mainly by introducing more environment information (such as wind direction or velocity) or other useful characteristics (such as repulsion or sensor denoise), so that the PSO algorithms can be faster out of local optimum or suitable for multi-odor source localization, and so on.
*The hybrid PSO algorithms* are obtained through concatenating and incorporating PSO with other swarm intelligence algorithms in order to solve PSO defects such as premature convergence, falling into local optimum, slow convergence speed, and so on.
*Other nature-inspired SI algorithms* mainly imitate the behavioral strategies of individuals in certain groups of organisms in nature through local perception and behavioral communication to achieve foraging, migrating, and escaping from natural enemies, to implement collaborative discovery, tracking, and localizing of odor sources in multi-robot systems.

The rest of this paper is organized as follows. Section Trending issues of robot OSL: An overview briefly discusses the trending issues of robot OSL. Section PSO and its variants in OSL: Origins and progress reviews the OSL methods based on PSO and its variants in detail. Section Other nature-inspired SI algorithms in OSL: Diversity and exploration reviews other swarm intelligence algorithms applied to robot OSL. Section Trends and challenges presents some trends and challenges in the future development of the robot active olfaction research. Finally, in last section, Conclusions are drawn.

## Trending issues of robot OSL: An overview

After more than two decades of development, the researches on mobile robots' odor source localization have gone through a process from simplicity to complexity, theory to application, broadly in the following aspects.

### Odor source: From single to multiple

Most robot OSL studies were conducted in the early years for single-point odor source environments. However, in a realistic situation, there may be point or surface odor sources with unknown numbers and locations emitting the same gas in the search area (Li et al., 2015).

Unlike the case of a single odor source, using multiple robots to locate multiple sources faces the following new problems.

#### How to maintain the exploration-exploitation balance in the search process

In earlier approaches, studies tended to emphasize exploitation, which would lead to premature convergence of the robot population once the extrema were found. Too many robots gathered in the same region tend to lose the diversity of individuals (Ghalia, 2008). However, if too much emphasis is placed on exploration, the algorithm will slow down the convergence. The dilemma is an extremely challenge faced by all multi-robot systems and swarm intelligence is advantageous but still need strictly assess in vastly different circumstances (Kwa et al., 2022).

#### How to search multiple odor sources in parallel

Traditional methods often take a sequential approach to locate multiple odor sources (Luo et al., 2008). In other words, only a single odor source can be located in a complete cycle (Zhang et al., 2015a), which affects the efficiency of odor source search.

#### How to avoid repeated searches for the same odor source

Such as how to issue a uniqueness statement for a specific odor source (Jatmiko et al., 2009b), determine whether the robot finds an existing odor source (Zhang J. et al., 2014), and how the robot swarm that finds an odor source continues searching for other odor sources.

To address the above shortcomings, some scholars have introduced the Niche technique into the OSL, which will be described in detail in the subsequent sections.

### Airflow environment: From diffusion to turbulence

The difference in the airflow environment signifies that the primary way of odor transmission is different. The current studies mainly focus on two types of environments: diffusion-dominated environment and turbulence-dominated environment.

In diffusion-dominated environment, where there is no turbulence, molecular diffusion becomes the determining factor for odor propagation, such as in the subsurface. Therefore, a simple gradient search can approach the odor source along the plume—most of the early studies used diffusion-dominated environment settings for simulation experiments.

In a turbulence-dominated environment, turbulent diffusion mainly affects odor transport, while molecular diffusion can be neglected (Smyth and Moum, 2001). The odor source forms a plume under the wind effect. The chaotic character of turbulence tends to cause random bending of the plume, producing vortices of different sizes. Large-scale vortices cause the entire plume to twist and wind, making tracking more complex (Yee et al., 1994), while small-scale vortices tear the plume into many filaments. Localized high concentration odor filaments can lead to localized abrupt concentration changes and concentration discontinuities (Kowadlo and Russell, 2008). In this environment, available wind speed/direction sensors can detect relatively accurate values, such as in general outdoor and ventilated indoor environments.

Another environment type is the weak fluid environment under turbulent dominance. In this environment, turbulence controls odors, but existing wind speed/direction sensors cannot obtain reliable data. For example, closed indoor environments do not exchange fluids with the outside world but generate weak convection through temperature differences. This environment is more demanding than others, and less relevant research is available.

In a turbulence-like dominant environment, discontinuous concentration gradients and sudden concentration shifts make it difficult for chemotaxis strategies to locate the odor source effectively anymore. Therefore, more sophisticated search strategies and algorithms are needed to escape the local optimum. The algorithms applicable to various airflow environments are described in more detail in the following section.

### Reactive principle: From chemotaxis to chemotaxis-anemotaxis

Chemotaxis and anemotaxis are two ways mobile robots utilize environmental information.

Chemotaxis refers to a method of mimicking organisms to rely on the concentration of the pheromone to reach the odor source. The robot uses the concentration measurements at different locations to calculate the concentration gradient and locates the odor source by climbing the odor concentration gradient. Plume dispersion is a steady process when the odor source is placed in a laminar (i.e., low Reynolds number) environment, which results in spatially coherent plume trajectories (Wang and Pang, 2021). In this case, the chemotaxis is effective. However, the odor concentration gradient in the actual case is not as smooth as expected because turbulence-dominated odor plumes are usually produced with vortices, and vortices of different sizes disrupt the shape of the plume with a smooth concentration gradient, which renders chemotaxis ineffective in this environment (Wang and Pang, 2021). Therefore, the utilization of wind information becomes an essential clue for odor source localization.

Anemotaxis is the process of some organisms in nature (e.g., dung beetles and silkworm moths) approaching an odor source against the wind or upstream in foraging and eventually locating the odor source. Inspired by this, earlier researchers have proposed to use wind information of organisms for OSL studies, mainly the Zigzag approach (Ishida et al., 1994), Silkworm moth algorithm (Russell et al., 2003), and Spiral Surge algorithm (Hayes et al., 2002). However, these studies are conducted in specific environments such as wind tunnels. The localization of odor sources cannot be effectively performed in a natural turbulent environment. In the last decade, the methods of anemotaxis have gradually changed from using wind information to using wind speed information combined with wind direction information. The upwind search ensures that the robot does not waste time searching in the wrong direction, and the wind speed helps the robot to adapt to the dynamic turbulent environment, which improves the search efficiency. Representative methods include the WUI and WUII methods (Jatmiko et al., 2016), adding upwind terms (Feng et al., 2019b), etc. The swarm intelligence algorithms utilizing both chemotaxis and anemotaxis will be described in detail in Sections PSO and its variants in OSL: Origins and progress and Other nature-inspired SI algorithms in OSL: Diversity and exploration.

### Agent: From individual to swarm

Embryonic single-robot active olfaction used a reactive principal to locating hazardous odor sources. Such robots acquire odor concentration signals through sensors, which trigger a preset sequence of behaviors for odor source search. The behavior of mobile robots often does not involve historical information, and the subsequent behavior of the robot is directly related to the sensor measurements in the present moment.

The benefits of using multiple robots in odor source localization are very intuitive. The OSL problem can be considered as the problem of finding the location of the maximum odor concentration in the target space. Therefore, it can be transformed into an optimization problem (Genovese et al., 1992). The global search in the target area can be completed faster and with a better multi-odor source search capability by using multiple agents. Compared to single robot, multiple robots require more “intelligent” algorithms to better plan robot swarm and avoid collisions with each other. Otherwise, multiple robots without mutual collaboration would instead cause degradation of search performance (Chen and Huang, 2019).

The following limitations are still present when using multi-robots to deal with the actual odor finding problem:

- Sensor cost limitations. The odor sensors employed in multi-robot systems cannot be too expensive. The response and recovery time of the sensors is slow, and the accuracy is not high.
- Working environment limitations. In the natural environment, there are often differences between the coordinate position values given by the robot positioning system and the actual position the robot is in, and the robots are prone to collision with each other;
- Sensor installation location limitations. Robots often can only detect the odor concentration at a vertical height.

Due to these factors, the odor concentration measurements are often inaccurate and contain noise. Under the influence of noise, the robot search process often appears to be prematurely stalled or meandered and sometimes even misled so that the robot is trapped in a pseudo-odor source location (Zhang Y. et al., 2014). In the subsequent sections, we will discuss how the improved PSO algorithm and the hybrid PSO algorithm weaken the effect of noise.

### Experimental validation: From computer simulation to real-life scenario

At present, three means are mainly used in active olfaction research, viz., simulation experiments, wind tunnel experiments, and field experiments to validate the OSL method. Experiments in a natural environment are the closest to the application scenarios. However, the experiments are difficult and costly to reproduce. They cannot meet the requirements of the high frequency of experiments and many changes in experimental conditions in the early research stage. Therefore, simulation was an essential tool in the initial research stage of OSL. Simulation can provide arbitrary configuration and reproducible virtual airflow environment for accurate simulation of odor plume and flow field, which helps to compare and verify the effect between different algorithms and between different improvements of the same algorithm and solve the problem of difficult reproduction and accurate comparison of odor experiments (Fan et al., 2017).

The distribution of the odor plume in the natural environment presents complex characteristics such as time-varying, interval, and multiple values (Meng et al., 2011), which are difficult to describe by constructing an accurate model. Therefore, most studies use relatively simplified numerical computational models. The plume models currently used in the OSL field mainly include the static Gaussian dispersion model (Ishida et al., 2005, 2006), Filament-based atmospheric dispersion model (Farrell et al., 2002), Lattice plume model (Balkovsky and Shraiman, 2002), Plume model based on CofinBox software package (Marques et al., 2006), etc.

## PSO and its variants in OSL: Origins and progress

### Standard PSO

Particle swarm optimization is an iterative optimization algorithm based on a simplified social population, inspired by the swarm behavior of a bird's flock: a flock of birds is searching for food randomly. There is only one piece of food in the area. All birds do not know where the food is, but they know the distance between their current position and the food. The best strategy in such a situation is to search the area around the bird that is currently closest to the food. Kennedy and Eberhart (1995) firstly proposed this algorithm for non-linear function optimization. In each iteration of PSO, every particle updates itself by tracking two “extremes” through experiences of individual and population which mean local optima and global optima, respectively. The PSO algorithm exhibits the weak computational properties of a single particle and the strong coordination of a population of particles. Therefore, it is considered as one of the most suitable optimization algorithms for multi-robot odor source localization.

The target search space is assumed to be *D*-dimensional in the standard PSO, and the position of the *i*th particle at time *t* can be represented as a *D*-dimensional vector ***X***_***i***_(*t*) = (*x*_*i*1_, *x*_*i*2_, …, *x*_*iD*_). The optimal position of the *i*th particle so far is ***P***_***i***_(*t*) = (*p*_*i*1_, *p*_*i*2_, …, *p*_*iD*_). The best position searched by the whole particle swarm so far is ***P***_***g***_(*t*) = (*p*_*g*1_, *p*_*g*2_, …, *p*_*gD*_), Equation 1 gives the velocity of the *i*th particle in the *D*-dimensional search space. The three terms on the right-hand side represent the motion direction of the original, the individual optimal, and the population optimal, respectively. Equation 2 updates particles' new position. Learning factors *c*_1_ *and c*_2_ are weights for options if the system is designed to tend to individual optimal or population optimal; *r*_1_ *and r*_2_ are two random numbers ranging from 0 to 1, *w* is the inertial factor, which tends to maintain the original direction with larger values.


(1) $$\begin{array}{l} V_{i}\left(t+1\right)=wV_{i}\left(t\right)+c_{1}r_{1}\left(P_{i}\left(t\right)-X_{i}\left(t\right)\right)\\ \;+c_{2}r_{2}\left(P_{g}\left(t\right)-X_{i}\left(t\right)\right) \end{array}$$

(2) $$\begin{array}{lll} X_{i}\left(t+1\right) & = & X_{i}\left(t\right)+V_{i}\left(t+1\right) \end{array}$$

Marques et al. (2006) first applied PSO to multi-robot collaboration to locate odor sources. In the plume discovery stage (which the author calls global search), they adopt a global random search strategy, integrating the repulsive force between agents and the biased crosswind motion. Once the local plume is found, it enters the local search stage, and the PSO algorithm is used for plume tracking. In this process, the fitness value of the particle is the concentration value of the pollutant where the particle is located. In the simulation experiment, the author compared the time of finding all odor sources in different atmospheric stability environments with three search algorithms: BRW local search, concentration gradient tracking, and particle swarm local search. The experiment showed that the PSO algorithm performs worst in a stable atmospheric molecular diffusion environment. However, in an unstable airflow environment with turbulence, i.e., the most realistic situation, the particle swarm search algorithm has an excellent advantage. Chen et al. (2017) proposed a multi-robot search method based on the PSO algorithm, incorporating a divergence search strategy for plume discovery and a mass flux divergence method for odor source declaration. The method was validated in a time-varying source environment with different ventilation environments, intensity variations, and obstructions.

The PSO algorithm can organize robots for search behavior but still has some drawbacks that need improvement, such as easily falling into local optima in non-ideal environments, inability to perform a multi-source search, and requiring a certain number of robots to ensure convergence speed. For better performance, scholars have made many improvements, which will be presented in the following subsection.

### Modified PSOs

Table 1 summarizes various of modified PSOs for odor source localization. The column names and their meanings are as follows: the aim of the study (Aim), the name of the modified algorithm (Algorithm), authors of the work (Author), the principal modification of the study (Modification), the reactive principle (Rec), the validation method (Val), the number of odor source being located (Odor), and the airflow environment (Air).

**Table 1:** Summary of the modified PSOs for odor source localization.

**Aim**	**Algorithm**	**References**	**Modification**	**Rec**	**Val**	**Odor**	**Air**
Escape from local optimum	WU-PSO	Jatmiko et al. (2007)	Introduces wind information	C/A	S	1	Dynamic turbulence environment with obstacles
	E-PSO	Ferri et al. (2007)	Introduces PI index and expands search area	C	S	1	Stable weak turbulence environment
	RW-PSO	Gong et al. (2012)	Introduces adaptive learning factors	C/A	S	1	Time-varying turbulence environment
	P-PSO	Li et al. (2008)	Introduces probability-based fitness	C/A	S	1	Stable turbulence environment
	P-PSO	Meng et al. (2011)	Introduces probability-based fitness	C/A	S	1	Time-varying turbulence environment
Adaptation to time-varying turbulent environments	UOA-PSO	Feng et al. (2019b)	Introduces wind-up terms and obstacle avoidance algorithms	C/A	S	1/N	Time-varying turbulence environment with obstacles (periodic and decay sources)
	CPSO	Feng et al. (2019a)	Integrates source identification algorithm and divergent search strategy	C/A	S/F	1	Time-varying turbulence environment (mechanical ventilation)
	URPSO	Feng et al. (2019c)	Adds a wind-up term and a random interference term	C/A	S/F	1	Stable turbulence environment
	ED-PSO	Feng et al. (2020)	Introduces the maximum concentration method and divergence search strategy	C	S/F	1	Stable turbulence environment
	P-PSO	Li et al. (2010)	Combines Bayesian inference with variable-universe fuzzy inference	C/A	S	1	Stable turbulence environment with obstacles
	Niche-PSO	Jatmiko et al. (2009a)	Introduces niche operations	C/A	S	1/N	Dynamic turbulence environment
	Charged PSO	Jatmiko et al. (2006a,b)	Introduces the mutual repulsive force	C	S	1	Dynamic turbulence environment with obstacles
	DR-PSO	Jatmiko et al. (2006a,b)	incorporates the change detection and responding mechanisms	C	S	1	Dynamic turbulence environment with obstacles
	FPSO	Lu et al. (2014a,b)	Introduces a non-linear damping term	C/A	S	1	Stable turbulence environment
Search for multiple odor sources	RS-PSO	Jatmiko et al. (2009b)	Adopts niche operations and parallel search	C/A	S/F	1/N	Dynamic turbulence environment
	Niching-PSO	Zhang J. et al. (2014)	Introduces niche operations	C	S	1/N	Stable turbulence environment with and without obstacles
Multi-robot collaboration	PSO-S-Consensus	Lu and Han (2010)	Adopts distributed coordination control scheme	C	S	1	Stable turbulence environment
	PSO-CCF	Lu et al. (2016)	Applies a collaborative control framework	C/A	S	1	Dynamic turbulence environment
	diverse-PSO	Jain et al. (2019)	Employs social spider optimization and formation operations	C	S	1/N	Stable turbulence environment
	APF-PSO	Fu et al. (2019)	Introduced an artificial potential field	C	S/F	1	Stable turbulence environment/outdoor ventilation environment
	MGC-PSO	Wang et al. (2017)	Introduces MCMC	C/A	S	1	Stable turbulence environment
Balance local search with global search	RR-GC-PSO	Yan et al. (2018)	Introduces a “request and reset” strategy	C/A	S	1	Air dispersion environment
Solve the noise problem of sensing input data	BB-PSO	Zhang Y. et al. (2014)	Adopts dynamical statistic method	C	S	1	Stable turbulence environment
Quickly complete the smoke plume discovery	DCS-PSO	Lu et al. (2013)	Applies decision-control system	C/A	S	1	Stable turbulence environment
Locate the odor source faster	SMC-PSO	Sinha et al. (2020)	Utilizes event-triggered sliding mode control	C/A	S	1	Stable turbulence environment

The letter C means chemotaxis, A means anemotaxis, N means multiple sources, S means simulation, and F means field experiment.

#### How agents escape from local optima

In the ventilated indoor environment, the odor plume fluctuates and is intermittently influenced by turbulence. Larger vortices may easily lead to lengthy local maxima. To solve this problem, Jatmiko et al. (2007) were the first to exploit the chemotaxis with wind information by introducing the angle θ between the wind vector ***W***(*t*) and the robot motion vector $V_{i}^{*}\left(t\right).$ Two WU-PSO methods are proposed for odor source localization in turbulent environments with obstacles. WUI is to set a forbidden area opposite the upstream direction of the turbulent flow, as shown in Equation 3, to ensure that particles move against the wind direction. WUII uses θ to calculate parameters χ_θ_ and then calculates the next updated position of particles through Equation 4.


(3) $$\begin{array}{lll} V_{i}\left(t+1\right) & = & \{\begin{array}{cc} 0 & \;if\;\theta <|\theta_{\text{forbidden }}|\\ V_{i}\left(t+1\right) & \;\text{Otherwise } \end{array} \end{array}$$

(4) $$\begin{array}{l} \begin{array}{ccc} V_{i}\left(t+1\right) & = & \chi_{\theta }\cdot V_{i}^{*}\left(t+1\right) \end{array} \end{array}$$

Ferri et al. (2007) proposed an explorative PSO algorithm (E-PSO) in an environment without strong winds, using an index based on the peak and average of historical odor concentrations as fitness and increasing the degree of exploration of the search area. Gong et al. (2012) proposed a modified PSO algorithm (RW-PSO) through introducing repulsive force and wind factors, which was validated in a time-varying simulation environment. Li et al. (2008) proposed a probabilistic particle swarm optimization (P-PSO) algorithm. The odor source probabilities estimated by Bayesian inference and variable universe fuzzy inference are used as expressions of the adaptation function. In P-PSO, they used wind information for constructing local probabilities of odor sources based on Bayesian inference, used concentration information for local probability maps of odor sources based on fuzzy inference, and then fused local probability distribution maps of odor sources from multiple robots to form a global raster probability map. Therefore, ***P***_***i***_(*t*) and ***P***_***g***_(*t*) in Equation 1 changed to the grid of the local maximum odor source probability up to the point in time and the global maximum odor source probability grid, respectively. Meng et al. (2011) proposed a plume estimation-searching framework based on P-PSO (Li et al., 2008) for slowly varying airflow environments (e.g., a slightly wandering large-scale advection-diffusion plume). It fuses the odor source probability distribution maps estimated independently by different robots at different times into a combined map based on the superposition of distances. The combined odor source probability distribution map expresses the adaptation function. The experimental simulation results show that it can approach the odor source with fewer robots.

#### How agents adapt to turbulent environments

**Figure 3:**
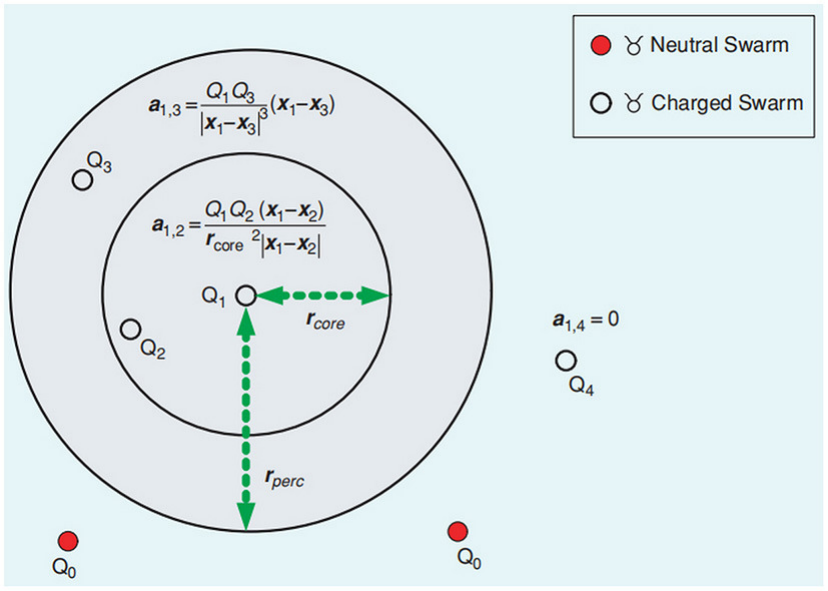
Interaction of the charged swarm robots (Jatmiko et al., 2007).

Most studies have been conducted in a mechanically ventilated, steady turbulent environment. However, winds in realistic scenarios tend to be time-varying, with unstable wind direction and speed, making the robot more susceptible to local optima due to large-scale vortices. In order to get out of the local optimum in a dynamically turbulent environment with obstacles, Li et al. (2010) further refined the proposed Probability-fitness-function based particle swarm optimization (P-PSO) algorithm, which integrates information on the size of odor concentration, concentration variation, and wind direction.


(5) $$\begin{array}{lll} P_{g}\left(t\right) & = & \arg\underset{m_{xy}}{\max}\left(p_{g_{nom}}\left(m_{xy},t\right)\right) \end{array}$$

(6) $$\begin{array}{lll} P_{i}\left(t\right) & = & \arg\underset{m_{xy}}{\max}\left(p_{L}\left(m_{xy}|z_{i,1:t}\right)\right) \end{array}$$

where *z*_*i*, 1:*t*_ is the detection event of the *i*th robot from moment 1 to moment t. *p*_*L*_(*m*_*xy*_∣*z*_*i*,1:*t*_) denotes the detection event of the raster *m*_*xy*_ passing the *i*th robot at time t. *p*_*g*_*nom*__(*m*_*xy*_, *t*) denotes the normalized odor source probability value of the raster *m*_*xy*_ at time t. Jatmiko et al. (2009a) proposed an modified PSO algorithm (Niche-PSO) incorporating the Niche technique, considering chemotaxis and anemotaxis. They later proposed two improved PSO methods (Jatmiko et al., 2006a,b), Detect and Respond PSO (DR-PSO) and Charged PSO. In the Charged PSO algorithm, the robot is divided into neutral and charged robots. The repulsive force between the charged robot and other charged robots obeys Coulomb's law, as shown in Figure 3, to ensure the diversity of some robots.

For a neutral robot, its position and velocity updating method are the same as Equations 1, 2; for charged robots, its position updating method refers to the Equation 2, and the velocity updating position method is like the Equation 7, *a*_*i*_(*t*) represents the total repulsive force of the robot at time *t*.


(7) $$\begin{array}{l} V_{i}(t+1)=wV_{i}(t)+c_{1}r_{1}(P_{i}(t)-X_{i}(t))\\ \;+c_{2}r_{2}(P_{g}(t)-X_{g}(t))+\underset{\text{repulsive force}}{\underset{︸}{a_{i}(t)}} \end{array}$$

Lu et al. (2014a,b) proposed a finite-time particle swarm optimization (FPSO) algorithm based on a continuous-time model and a discrete-time version of the FPSO algorithm. In order to make particles converge in a finite time interval, a non-linear damping term is introduced. The simulation results show that the discrete-time version of FPSO can successfully locate the odor source.

In order to adapt to the dynamic turbulence environment, Feng et al. (2019b) proposed a PSO algorithm with an upwind term and obstacle avoidance algorithm (UOA-PSO), which adds an upwind term to the standard PSO algorithm to utilize wind information for locating time-varying attenuation sources and time-varying periodic sources with higher success rate than standard PSO and WUII methods. In the algorithm, an upwind velocity $V_{i}^{u}\left(t\right)$ at moment *t* is added to the Equation 1 for the *i*th robot position and velocity, as shown in Equation 5, where *c*_3_ reflects the effect of airflow on the robot velocity and *r*_3_ is a random number in [0, 1].


(8) $$\begin{array}{l} V_{i}\left(t+1\right)=wV_{i}\left(t\right)+c_{1}r_{1}\left(P_{i}\left(t\right)-X_{i}\left(t\right)\right)\\ \;+c_{2}r_{2}\left(P_{g}\left(t\right)-X_{i}\left(t\right)\right)+c_{3}r_{3}V_{i}^{u}\left(t\right) \end{array}$$

Subsequently, Feng et al. (2019a) proposed a comprehensive particle swarm optimization (CPSO) method that combines a source identification algorithm and a strategy to escape from local extremal regions based on the previous UOA-PSO method (Feng et al., 2019b). The effectiveness of method was realistically verified by three robots in a typical time-varying airflow environment and compared with standard PSO and WUII methods in an experimental setting, concluding that the CPSO has higher source localization efficiency. To avoid successive local extrema, Feng et al. (2019c) proposed another multi-robot sniffing approach (URPSO) based on an adaptive particle swarm optimization algorithm. This method incorporates an upwind term and a random interference term, and combines a divergence search strategy and a maximum concentration method. Feng et al. (2020) further proposed a modified PSO algorithm (ED-PSO) introducing extremum disturbance factors, which can be independent of any airflow information. The individual optimal position ***P***_***i***_(*t*) and the global optimal position ***P***_***g***_(*t*) are updated as follows:


(9) $$\begin{array}{l} P_{i}{\left(t\right)}^{\prime }=P_{i}\left(t\right)+c_{3}V_{max}P_{i}^{r} \end{array}$$

(10) $$\begin{array}{l} P_{g}{\left(t\right)}^{\prime }=P_{g}\left(t\right)+c_{4}V_{max}P_{g}^{r} \end{array}$$

where *V*_max_ is the maximum magnitude of the velocity vector of each robot, namely, the maximum step length of each robot. $P_{i}^{r}$ and $P_{g}^{r}$ are two disturbance vectors uniformly distributed in [−1, 1]. *c*_3_ and *c*_4_ are dimensionless parameters that reflect the disturbance magnitudes of two disturbance vectors on ***P***_***i***_(*t*) and ***P***_***g***_(*t*), respectively. The method consists of three core algorithms: an improved PSO algorithm introducing extreme value disturbance factors, a maximum concentration method for plume source declaration, and a dispersion search strategy for plume finding and escape from local extreme value regions. The robustness of method was demonstrated in an experimental environment with indoor mechanical ventilation.

#### How agents find multiple odor sources

Jatmiko et al. (2009b) proposed a modified PSO algorithm (RS-PSO), where they used niche and parallel search characteristic to deal with multi-peak and multi-source problems. In addition, a range subgroups method was introduced to improve the efficiency of the search capability. Zhang J. et al. (2014) proposed a Niching PSO-based method for cooperative localization of odor sources by robots. A random search algorithm is used to search for the plume in the global search phase. After finding the plume, the particle and the particles within the neighboring radius R form a niche, and all particles inside the niche will track the same plume while different niches are applied to locate different odor sources simultaneously. To locate different odor sources efficiently, a dynamic adjustment strategy of niche size based on the degree of aggregation and a merging strategy based on optimal similarity are applied. In addition, practical conditions such as the sampling/recovery time of the sensor and the speed limit of the robot are also considered.

The particle velocity update method is as follows:


(11) $$\begin{array}{l} V_{k}\left(t+T\right)=wV_{k}\left(t\right)+c_{1}r_{1}\left(P_{k}\left(t\right)-X_{k}\left(t\right)\right)\\ \;+c_{2}r_{2}\left(P_{g}\left(t\right)-X_{g}\left(t\right)\right) \end{array}$$

The difference with Equation 1 is that *k* = 1, 2, …, *N*_*i*_, *N*_*i*_ in Equation 11 is the size of the ecotone, and *T* is the sum of sensor sampling and recovery time. The method was simulated in a naturally ventilated indoor environment with obstacles, and the results show that the algorithm can locate several odor sources simultaneously with a high success rate.

#### How to coordinate agents

In previous literature, robots were often treated as mass points. However, in reality, they are not. In order to coordinate the movement between multiple robots and prevent collisions between robots, Lu and Han (2010) designed a distributed coordination control method (PSO-S-Consensus) consisting of an artificial layer and a control layer. Trajectory level has been employed in the proposed control scheme. Lu et al. (2016) designed a cooperative control framework for the PSO algorithm implementing a collective decision mechanism for the ordered motion behavior of the particles. The cooperative control framework consists of a position coordination term, a velocity coordination term, and a motion direction coordination term. Based on this framework, the PSO-CCF algorithm was proposed for the OSL problem, which can coordinate the relative positions between robots and ensure the orderly motion of robots to avoid collision and capture time-varying plumes. Jain et al. (2019) proposed a cooperative multi-robot localization method based on diverse-PSO and introduced four kinds of robot formation operation. Five different robot behaviors are added in the plume tracking phase. In the three-dimensional turbulent simulation environment with and without errors, the experimental results show that the diverse-PSO algorithm is superior to the Modified PSO, Niche-PSO, and R-PSO (Yan et al., 2018).

Searching with swarms of unmanned aerial vehicles (UAVs) can be regarded as odor source localization in three-dimensional space, allowing odor source detection in more complex contaminated environments than employing ground-based robotic teams to search for odor sources. Traditionally, particle swarm optimization algorithms treat robots as prime points, and this concept obviously cannot be applied to UAV search. Fu et al. (2019) proposed an APF-PSO algorithm using an artificial potential field method for generating safe and smooth UAV swarm flight paths, which improves the safety of the algorithm. Meanwhile, adaptive inertia weights and exclusion zones are introduced to help particles escape from the local optimum. Simulation experiments show that the adoption of this algorithm has a significant impact on the search success rate and efficiency.

#### How to improve other performances

To better balance local search with global search at different stages, Yan et al. (2018) introduced a “request and reset” strategy into the Guaranteed Convergence PSO (Van den Bergh and Engelbrecht, 2002) and Dissipative PSO (Xie et al., 2002) and both added a modified learning factor and inertia weight, to form two modified PSOs (RR-GC-PSO and MD-PSO). The “Request and Reset” strategy can remove the low-fitness particles and re-incorporate them into the global search to enhance the algorithm's global search capability. The simulation results show that the RR-GC-PSO algorithm converges faster and has a higher success rate for robots in a large environment with small population size. The velocity update equation of robot in “optimal group” in RR-GC-PSO, as shown in Equation 12, is quite different from that in standard PSO.


(12) $$\begin{array}{l} V_{i}\left(t+1\right)=wV_{i}\left(t\right)-P_{i}\left(t\right)+P_{\varphi g}+\rho \left(1-2r_{2}\right) \end{array}$$

where ***p***_*φ**g***_ is the global optimal position detected by robot φ, and ρ is a scaling factor related to the size of the search area.

To improve the localization accuracy of the algorithm, Wang et al. (2017) proposed a combined approach using both static and mobile sensors, using modified genetic algorithms (MGA), Markov Chain Monte Carlo (MCMC), and MGC-PSO algorithms. In this study, OSL was divided into three stages: static sensors for data acquisition, a rough calculation of the search area, and a mobile robot swarm for odor source localization. First, the MGA algorithm estimates a possible source of leakage as the initial sampling point for MCMC sampling. The MCMC then calculates search areas that are likely to contain odor sources. Finally, the mobile robot uses the MGC-PSO algorithm to locate the leakage source accurately. Simulation results show that the method can locate the odor source within one meter.

The odor concentration sensors measured by robots during source localization commonly contain noise. Zhang Y. et al. (2014) proposed a collaborative multi-robot search method based on Bare-Bones PSO (BB-PSO) to solve this problem. Dynamic statistics estimates the noise intensity of odor concentration measured by the robot, and the fitness value of particles in a noisy environment is expressed by interval number. Particles' position update based on the defined particle probability dominance relation. Let the measured odor concentration value of the robot at position ***X***_***i***_(*t*) be *f*(*a*_*j*_), and the intensity of the measured odor concentration value of the robot at the current position ***X***_***i***_(*t*) affected by noise is estimated as follows:


(13) $$\begin{array}{l} \rho_{i}\left(t\right)=\frac{1}{m}\overset{m}{\underset{j=1}{\sum }}|f\left(a_{j}\right)-0.5\left(f\left(a_{j-1}\right)+f\left(a_{j+1}\right)\right)| \end{array}$$

where *t* is the number of iterations, the absolute difference is calculated by the measured odor concentration value and the estimated value (the average of the measured odor concentration values at adjacent positions on both sides of position *j*). The true odor concentration value of the location ***X***_***i***_(*t*) of will fall with a high probability in the interval [*f*(***X***_***i***_(*t*))−ρ_*i*_(*t*), *f*(***X***_***i***_(*t*))+ρ_*i*_(*t*)]. This interval is used as the fitness of the particles in the current position for particle update, and the probability interval method is used to avoid the influence of noise. In the turbulent simulation environment, the experimental results show the excellent performance of the method.

To enable robots to find odor cues quickly, Lu et al. (2013) introduced finite-time parallel and cyclic controllers for parallel motion and circular motion of robot groups and designed a PSO method based on two-layer decision-control system (DCS-PSO) for a hierarchical response. The wind information is introduced in the decision layer. The convergence of the finite-time algorithm is analyzed by using the Lyapunov method, and simulation experiments verify the effectiveness of the proposed method.

In order to locate the odor source faster for effective decision making under communication and computational resource constraints, Sinha et al. (2020) proposed a PSO algorithm based on a hierarchical cooperative control strategy (SMC-PSO) including a group decision layer and a robust collaborative control layer. In the group decision layer, the modified PSO, which introduces odor concentration information and wind information, is used to make a group decision and send it to the robust collaborative control layer. The robust collaborative control layer implements a sliding mode controller in an event-triggered fashion, which ensures that the robot can converge to the equilibrium point in a limited time.

### Hybrid PSOs

The simulated annealing algorithm (SA) is derived from the simulation of the solid annealing process (Kirkpatrick et al., 1983) and is a kind of heuristic Monte Carlo method. It can be applied to optimization problems, namely to locate an excellent approximation to the global minimum of a given function in a vast search region. To prevent premature convergence of the particle swarm, Gong et al. (2012) proposed a SA-RWPSO algorithm that combines SA algorithm and multiple dynamic factors (here RWPSO is PSO based on repulsive force and wind). In the standard PSO algorithm, if the current position of a particle is better than the historical best position ***P***_*best*_, the current position will become the new best position ***P***_*best*_. The direct replacement strategy will result in a tendency for the particles to converge to the optimal position ***P***_*best*_ prematurely, and particles are more likely to lose diversity. In SA, the algorithm can accept deteriorating solutions with a certain probability and accept optimized solutions. The particle will accept the new position ***X***_***i***_(*t*+1) of the *i*th particle at moment *t* with probability δ_*pro*_ in the case that ***X***_***i***_(*t*+1) is inferior to ***P***_***i***_(*t*). Moreover, δ_*pro*_ will decrease as the number of temperature iterations increases. This indicates that particle diversity can be preserved in the initial stage of the algorithm, and convergence is guaranteed in the later stages, which represents that the simulated annealing algorithm can both jump out of the local optimum and converge to the vicinity of the global optimum solution. Therefore, the SA algorithm is feasible for solving the OSL problem with multi-polar concentration functions indoors. Simulation results in four typical scenarios show that the proposed algorithm can guide the multi-robot system to quickly and accurately locate the odor source.

Also, to prevent particles from being trapped locally, Zhang et al. (2015b) proposed a refined hybrid PSO algorithm that combines two bacterial foraging optimization (BFO) operations for odor source localization in robot swarms. In the proposed algorithm, the authors integrate the convergence operation into the PSO algorithm to guide the robot to track the plume and employ the elimination-dispersion operation to avoid particles falling into local minima. In standard PSO algorithm, if the current position of a particle is very close to the individual optimal position and the globally optimal position in the late iteration, the velocity of all particles will drop to very small, or even zero, which will cause all particles to stop updating prematurely and the algorithm will converge to a false local optimum. Hence, the authors adopt a better approach by proposing an improved BFO elimination and diffusion method that searches for a better solution near the wrong local optimum instead of stopping the movement. In the BFO-PSO algorithm, the particle swarm, after being updated by Equations 1, 2, will be updated again using the chemotactic equation as:


(14) $$\begin{array}{l} X_{i}\left(t+1\right)=X_{i}\left(t\right)+S\left(i\right)\frac{V_{i}\left(t+1\right)}{\sqrt{V_{i}{\left(t+1\right)}^{T}V_{i}\left(t+1\right)}} \end{array}$$

where *S*(*i*) is the unit travel length of the agents. From Equation 14 it can be seen that the agent will move further along the velocity direction. This is followed by an improved elimination and dispersion of BFO to prevent convergence to localization, as follows:


(15) $$\begin{array}{l} X_{i}\left(t+1\right)=\{\begin{array}{c} X_{i}\left(t\right)+S\left(i\right)\frac{\Delta \left(i\right)}{\sqrt{{\left(\Delta \left(i\right)\right)}^{T}\Delta \left(i\right)}}\;if\;\text{rand }\left(\;\right)\;<P_{ed}\\ X_{i}\left(t\right),\;\text{otherwise } \end{array} \end{array}$$

where Δ(*i*) is the random direction vector and *P*_*ed*_ is the probability of elimination and dispersion.

To balance exploration and exploitation in multi-robot target search, Jain et al. (2018) used the gray wolf optimization (GWO) in concatenation with the PSO algorithm. In the GWO algorithm, the prey's surrounding behavior will help keep the robot within the plume area. Two GWO-PSO and PSO-GWO plume tracking algorithms are proposed by the concatenated method for multi-robot collaboration and prevention of premature local convergence. However, this method cannot locate multiple odor sources in parallel.

Nevertheless, the BFO-based PSO algorithm, the GWO-based PSO algorithm, and most of the hybridization algorithms require the tuning of control parameters to achieve the best efficiency. This represents a difference in the search efficiency of the algorithms in different environments. Gaurav et al. (2020) proposed a parameter-free hybrid teaching learning particle swarm algorithm (HTLPSO). In this method, the teaching phase of TBLO is merged with the PSO algorithm, which helps to improve the computational speed and efficiency. Simulation results in single odor source and multiple odor source environments demonstrate the search effectiveness of HTLPSO and its high accuracy convergence even with a small number of agents.

Table 2 summarizes various of hybrid PSOs for odor source localization. The column names and their meanings are as follows: the name of the hybrid algorithm (Algorithm), the purpose of the study (Aim), authors of the work (Author), the algorithm combined with PSO (Com), the reactive principle (Rec), the validation method (Val), the number of odor source being located (Odor), and the airflow environment (Air).

**Table 2:** Summary of the hybrid PSOs for odor source localization.

**Algorithm**	**References**	**Aim**	**Com**	**Rec**	**Val**	**Odor**	**Air**
RWPSO	Gong et al. (2012)	Prevents premature convergence of the swarm	SA	C/A	S	1/N	Stable turbulence environment
BFO-PSO	Zhang et al. (2015b)	Prevents particles from being trapped locally	BFO	C	S	1	Stable turbulence environment
GWO-PSO	Jain et al. (2018)	Balances between exploration and exploitation of the workspace.	GWO	C/A	S	1	Stable turbulence environment
PSO-GWO	Jain et al. (2018)	Balances between exploration and exploitation of the workspace.	GWO	C/A	S	1	Stable turbulence environment
HTLPSO	Gaurav et al. (2020)	Improves search performance in MOS and SOS environments	TLBO	C	S	1/N	Stable turbulence environment

The letter C means chemotaxis, A means anemotaxis, N means multiple sources, S means simulation, and F means field experiment.

## Other nature-inspired SI algorithms in OSL: Diversity and exploration

Table 3 summarizes various of nature-inspired SI algorithms applied to odor source localization. The column names and their meanings are as follows: the name of SI algorithm (Algorithm), authors of the work (Author), the principal operation of the work (Operation), the reactive principle (Rec), the validation method (Val), the number of odor source being located (Odor), and the airflow environment (Air).

**Table 3:** Summary of other nature-inspired SI algorithms applied to odor source localization.

**Algorithm**	**References**	**Operation**	**Rec**	**Odor**	**Val**	**Airflow**
CSA	Wang et al. (2016)	Sets up a restricted position	C/A	1	S	Stable turbulent environment
	Wu and Wang (2021)	Introduces the concept of territory	C	1/N	S	Stable turbulent environment
ACO	Meng et al. (2006)	Combines with GA	C	1	S	Stable turbulent environment
	Zou and Luo (2008) and Zou et al. (2009)	Adds an odor source verification strategy	C	1/N	S	Stable turbulent environment
	Meng et al. (2010)	Adds upwind search algorithm	C/A	1	S/F	Time-varying turbulent environment
	Cao et al. (2013)	Introduces selective olfaction and continuous source statements	C/A	1/N	S	Outdoor natural turbulent environment
	Che et al. (2018)	Shares global pheromone distribution map	C/A	1	S	Time-varying turbulent environment
GSO	Krishnanand and Ghose (2005) and Krishnanand et al. (2006)	Introduces variable local decision fields	C	1/N	S	Dynamic turbulent environment
	Zhang et al. (2011)	Introduces the forbidden area	C	1/N	S	Constant diffusion environment
GWO	Shen et al. (2021)	Employs vision sensors	C/V	1	S/F	Constant diffusion environment
WOA	Yang et al. (2019)	Introduces wind tendency	C/A	1	S	Time-varying turbulent environment
	Jiang et al. (2022) and Zhou et al. (2022)	Introduces wind information	C/A	1	S/F	Time-varying turbulent environment
AEO	Fu et al. (2021)	Introduces discrete wind system	C/A	1	S	Stable turbulent environment

The letter C means chemotaxis, A means anemotaxis, V means vision information, N means multiple sources, S means simulation, and F means field experiment.

### Cuckoo search optimization

Cuckoo search optimization (CSA; Yang and Deb, 2009) is inspired by the parasitic brooding behavior of cuckoo populations and the Lévy flight behavior during nest migration. The cuckoo search algorithm has the advantages of simplicity, few parameters, and easy implementation. In dealing with complex optimization problems, there is no need to set many parameters in CSA. In fact, in addition to the population number *n*, there is only one parameter *P* in CSA. Lévy flight and preference random walk are two critical components of the cuckoo search algorithm, which makes the CSA better global searchability and have solid local searchability. They can get better results for almost all optimization problems.

Wang et al. (2016) proposed a method for locating odor source based on the CSA algorithm. In the plume discovery phase, this method introduces the concept of a taboo position. The distance of the robot's new target location should be greater than the distance between the taboo position and the distance threshold. In the plume tracking phase, a master-slave robot search mechanism is employed. It uses the robot that detects the highest odor concentration among all robots to guide the other robots to search for the odor source in its upwind direction. Let the serial number of the master agent at the *g*th iteration be *N*_*best*_, and the location be *X*_*best*_. At the *g*+1th iteration, all agents will be updated at the position *X*_*best*_ of the master agent with Equation 16:


(16) $$\begin{array}{l} X_{g+1,i}=X_{g,best}+L_{i},\left(i=1,2,\cdots \;,N\right) \end{array}$$

where *X*_*g*+1, *i*_ represents the updated position of the *i*th robot at the *g*+1st iteration, *L*_*i*_(*i* = 1, 2, …, *N*) is the step vector whose modulus *d* obeys the Lévy distribution and *N* is the number of robots. To improve the search efficiency, the method incorporates upwind search and a local odor concentration optimization mechanism. In a simulated environment with indoor ventilation, it is verified that the method can guide multiple robots to search for odor sources faster compared with the U-ACO algorithm.

In order to further improve the cuckoo algorithm source finding efficiency with an extensive range search for multiple scent sources, Wu and Wang (2021) proposed an improved CSA. The forbidden areas were introduced as the cuckoo's territory to prevent the cuckoo from falling into local optimal positions. When a certain number of cuckoos are close to the same scent source, the best local cuckoo in the area acts as the center of the area, declared as the forbidden area. The area is no longer repeatedly searched by other robots. The concept of forbidden area merging was introduced to prevent repeated discovery of the same odor source. Simulation results show that the method can accurately locate multiple odor sources in a Gaussian diffusion model environment.

### Ant colony optimization

Ant colony optimization (ACO) is proposed to simulate the foraging behavior of ant colonies in nature (Dorigo et al., 1996). It has the characteristics of positive feedback, parallel computing, good robustness, etc. Ants will continuously secrete pheromones during their out-feeding walk, and as the pheromones accumulate, most ants later will follow this shortest path. The traditional ant system consists of three main parts: initialization, solution construction, and pheromone update. Through the accumulation of pheromones, its search results have strong robustness, but there are still many shortcomings. For example, in the early stage of global search, the uniform distribution of pheromones may slow down the iteration speed and make ants search blindly. Meanwhile, the algorithm is prone to fall into local optimal solutions. Meng et al. (2006) were the first to use an improved ACO combined with a genetic algorithm for solving the OSL problem: in the iterative process, the genetic algorithm is first used for local search, then the ACO for global search, and finally pheromone updates. In the global search phase, under appropriate conditions, robot *i* performs a global search with probability *p*(*i, j*) for the region where robot *j* is located for a certain number of steps as follows:


(17) $$\begin{array}{l} p\left(i,j\right)=\frac{\tau^{\alpha }\left(j\right)e^{d_{ij}\beta }}{\overset{m}{\underset{j=1}{\sum }}\tau^{\alpha }\left(j\right)e^{d_{ij}\beta }},\;\left(j=1,2,\cdots \;,m\right) \end{array}$$

where *d*_*ij*_ = *C*(*j*)−*C*(*i*) is the difference between robot *j* and the locally optimal concentration in the region where robot *i* is located, τ^α^(*j*) is the pheromone of robot *j*, α and β are the weight parameters, and *m* is the number of robots. After all robots have completed the global search, if *n* robots move toward the region where robot *i* is located and increase their concentration, the pheromone in the region where robot *i* is located will be updated as follows:


(18) $$\begin{array}{l} \tau \left(i\right)=\lambda \cdot \tau \left(i\right)+\overset{n}{\underset{j=1}{\sum }}C\left(j\right) \end{array}$$

where λ is a coefficient describing the degree of pheromone decay, in (0, 1). This method can find the location of an odor source with fewer iterations, but the effectiveness of the method for searching multiple odor sources has not been verified. Zou and Luo (2008) and Zou et al. (2009) proposed an improved search strategy for the ACO by redefining the pheromone and heuristic functions, adding two search modes, local traversal search and global random search, and including a validation procedure for recording multiple odor sources in a single iteration. Simulation results demonstrate the effectiveness of the improvement. However, the method causes the robot to repeatedly converge to the searched odor sources, which reduces the search efficiency of multiple odor sources. Meng et al. (2010) will propose an improved ant colony algorithm (US-ACO) combined with an upwind search algorithm for a time-varying indoor odor environment and validate its effectiveness in a natural indoor turbulent environment. Further, Cao et al. (2013) introduced the concept of selective olfaction based on the US-ACO algorithm and proposed an asynchronous ant colony optimization algorithm (SoACO) for fast localizing multiple odor sources. Specifically, the concentration sensor will selectively stop working during the search to jump out of the local optimum and the multi-source search. This approach successfully found multiple odor sources continuously within fewer robots in the simulation experiments. Che et al. (2018) divided the robots into two groups according to the pheromone concentration to perform the tasks of upwind search and improved ant colony algorithm search, respectively. In the process of global pheromone update, the influence of time-varying wind field on odor plume was considered, in which the wind speed determines the update frequency. Simulation results show that the algorithm has a high success rate and search efficiency.

### Glowworm swarm optimization

**Figure 4:**
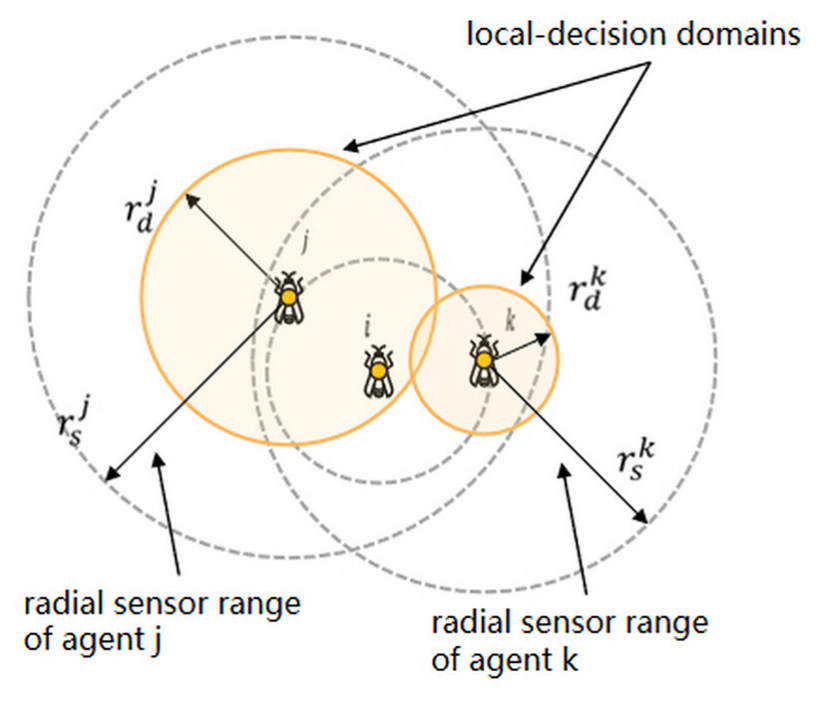
Glowworm *i* is in the sensor range of (and is equidistant to) both *j* and *k*. However, *j* and *k* have different local-decision domains, and only *j* uses the information of $i\;\left(r_{d}^{k}<r_{d}^{j}<r_{s}^{k}<r_{s}^{j}\right)$. Figure modified from Krishnanand et al. (2006).

Glowworm swarm optimization (GSO) can be employed to compute multiple optimal values of multimodal functions simultaneously. The algorithm was originally proposed by Krishnanand and Ghose (2009) as a variant of the ACO algorithm, but several significant modifications exist. Like the ACO, where each motion area is associated with a pheromone value, the agents in the glowworm algorithm also carry a luminous quantity. Agents are considered to be glowworms, which emit light of an intensity proportional to the associated quantity of luminescence and have a circular sensor range. The glowworm *k* has a circular sensor range $r_{s}^{k}$, to define the variable local decision domain $r_{d}^{k}\left(0<r_{d}^{k}<r_{s}^{k}\right)$ for computing motion (as shown in Figure 4). As with the ACO, each agent *i* has a certain probability *p*_*j*_(*t*) to move to the region where neighbor *j* is located, and the discrete-time model for an agent position update is as follows:


(19) $$\begin{array}{l} X_{i}\left(t+1\right)=X_{i}\left(t\right)+s\left(\frac{X_{j}\left(t\right)-X_{i}\left(t\right)}{\left\|X_{j}\left(t\right)-X_{i}\left(t\right)\right\|}\right) \end{array}$$

(20) $$\begin{array}{l} s=\{\begin{array}{c} \delta ,\;if\;d\left(i,j\right)\geq \delta \\ d\left(i,j\right),\text{ otherwise } \end{array}\; \end{array}$$

where *d*(*i, j*) denotes the distance between robot *i* and robot *j*. To simulate the decay of fluorescein with time, the rule for updating the fluorescein of robot *j* at moment *t* is as follows:


(21) $$\begin{array}{l} \ell_{j}\left(t+1\right)=max\left\{0,\left(1-\rho \right)\ell_{j}\left(t\right)+\gamma J_{j}\left(t+1\right)\right\} \end{array}$$

where *J*_*j*_(*t*) denotes the value of the luciferin level of agent *j* at moment *t*, γ is the proportionality constant used to increase the luciferin level, and ρ is the luciferin decay constant (0 < ρ < 1 ).

Benefiting from the property that the GSO can find the optimal solution of multiple optimal continuous functions (Krishnanand and Ghose, 2005; Krishnanand et al., 2006; Zainal et al., 2013) used the GSO for solving the multi-source localization problem. Variable local decision domains are also applied to glowworms. The variable local decision domain update formula for glowworm *i* at time *t*+1 is as follows, and the explicit threshold parameter *n*_*t*_ is introduced to attenuate the oscillatory behavior of the decision domain:


(22) $$\begin{array}{l} r_{d}^{i}\left(t+1\right)=\{\begin{array}{cc} r_{d}^{i}t+\beta_{1}|N_{i}\left(t\right)|, & \;\text{if }|N_{i}\left(t\right)|\leq n_{t}\\ r_{d}^{i}t-\beta_{2}|N_{i}\left(t\right)|, & \;\text{otherwise } \end{array} \end{array}$$

where β_1_ and β_2_ are constant parameters. This algorithm proved its superiority over the ACO in a multi-source environment through simulation and field experiments. Zhang et al. (2011) proposed a multi-robot cooperation strategy based on a modified GSO (M-GSO). The concept of forbidden area was introduced, and they argued that the forbidden area setting has two goals. One is to ensure that the robots inside the forbidden area are released so that they have the opportunity to find another odor source. The other is to ensure that robots outside the forbidden area do not repeatedly locate this odor source. Simulation results in a benchmark environment of multiple odor source localization show that M-GSO can find all indoor odor sources with fewer iterations than the traditional GSO algorithm.

### Gray wolf optimization

Gray wolf optimization is a population intelligence optimization algorithm inspired by gray wolf populations (Mirjalili et al., 2014). The method mimics the strict four social classes and hunting behaviors within the gray wolf population. Different social classes of gray wolves will be responsible for decision-making, training, hunting, scouting and patrolling, and other divisions of the wolf pack.

**Figure 5:**
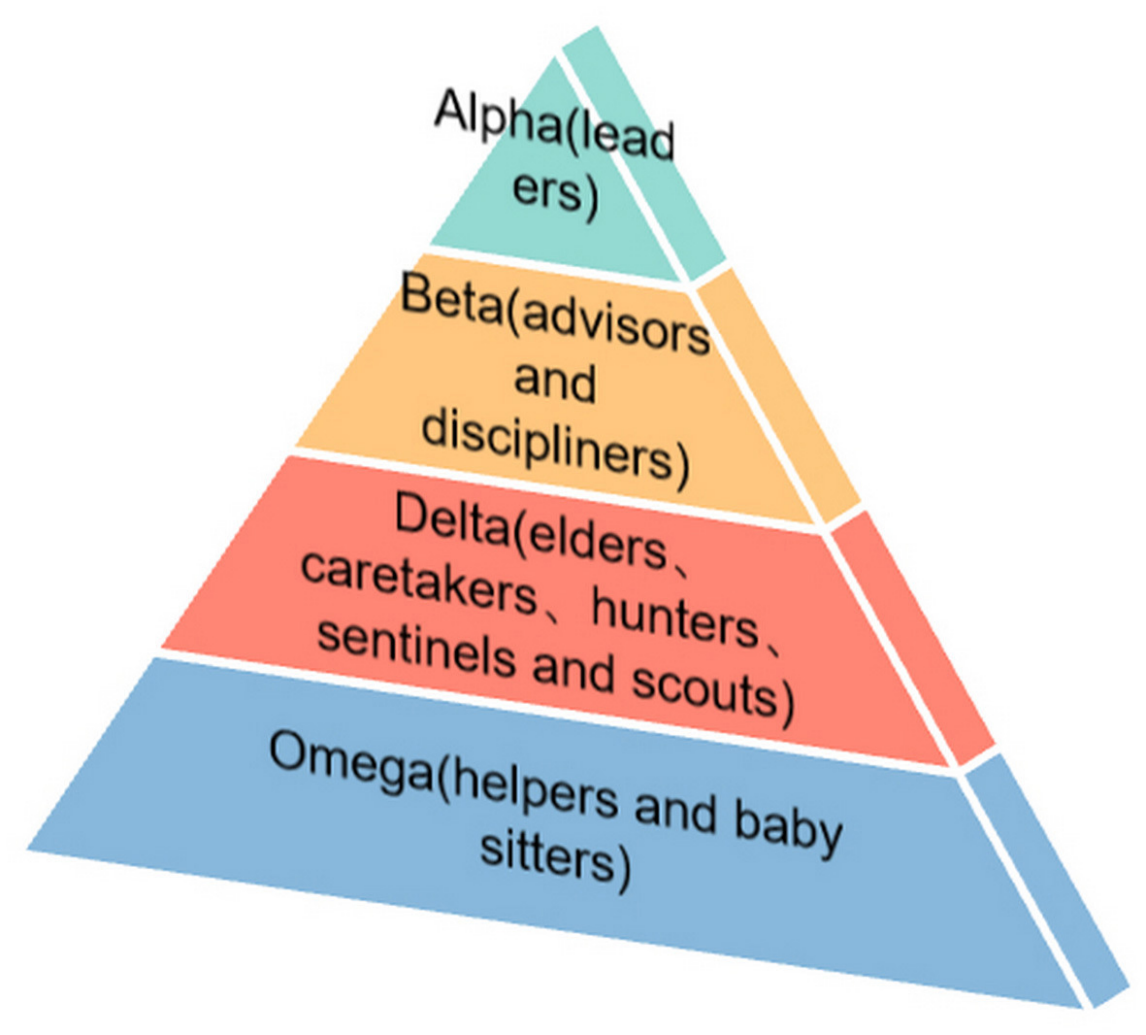
Schematic diagram of the hierarchy of GWO.

To improve the speed of early plume search and the reliability of plume tracking, Shen et al. (2021) made the autonomous mobile robot simulate the social mechanism and hunting behavior of gray wolf populations to plan the robot path and find the optimal value in plume tracking. Using the four different classes of gray wolf groups, a hierarchical structure suitable for robot search is constructed. The four levels decrease from top to bottom as alpha, beta, delta, and omega, respectively (Figure 5). The field experimental results show that this method can successfully locate the leakage odor source in some three-dimensional diffusion environments, but only a single robot is employed. Mamduh et al. (2018) tried to propose a cooperative multi-robot strategy based on GWO that uses the prey search phase of gray wolves to track plumes. Before approaching the odor source, the robots will surround the odor source, thus avoiding the searched odor source being different from the actual location, as in other algorithms. Robots are divided based on social hierarchy in the process. Robots with the three best solutions are defined as *alpha*(α), *beta*(β), and *delta*(δ) based on the sampled concentration data. The other robots are assumed to be *omega* (ω). The ω robots generally are guided by the α, β, and δ robots. After the wolves have surrounded the searched scent source, the wolves will hunt and update the location as Equations 23–25:


(23) $$\begin{array}{l} \begin{array}{c} D_{\mu }=|C_{1}\cdot X_{\mu }-X|\\ D_{\beta }=|C_{2}\cdot X_{\beta }-X|\\ D_{\delta }=|C_{3}\cdot X_{\delta }-X| \end{array} \end{array}$$

(24) $$\begin{array}{ll} X_{1} & =X_{\alpha }-A_{1}\cdot D_{\alpha }\\ X_{2} & =X_{\beta }-A_{2}\cdot D_{\beta }\\ X_{3} & =X_{\delta }-A_{3}\cdot D_{\delta } \end{array}$$

(25) $$\begin{array}{l} X\left(t+1\right)=\frac{X_{1}+X_{2}+X_{3}}{3} \end{array}$$

where ***D***_***α***_ denotes the encirclement vector of the robot α, ***X***_***α***_ denotes the target position vector of the robot α, ***X*** denotes the movement vector of the robot, and A_*_ and ***C*** are the computed coefficient vectors.

Moreover, in the literature already reviewed in the previous section, Jain et al. (2018) proposed a multi-robot system with two algorithms in tandem. It achieved good results by concatenating the gray wolf optimizer with the particle swarm optimizer.

### Whale optimization

Whale optimization (WOA) finds the optimal solution by imitating the search process of whales catching food in the ocean (Mirjalili and Lewis, 2016). The algorithm has the characteristics of a simple structure and few parameters. It is faster and more accurate than standard PSO and genetic algorithm in solving multivariate functions and can jump out of local optimization (Mirjalili and Lewis, 2016).

To rapidly locate indoor time-varying pollution sources, Yang et al. (2019) pioneered standard WOA (SWOA) for multi-intelligence active olfaction and proposed an improved WOA method by introducing an upwind term. In the plume tracking phase, the WOA algorithm simulates the hunting behavior of the humpback whale by three operators, namely, the encircling prey operator, bubble-net attacking operator, and searching for prey operator. The operation of the encircling prey operator is similar to the GWO algorithm. For the robot position at the moment *t*+1 will be updated as Equation 26:


(26) $$\begin{array}{l} \begin{array}{c} X\left(t+1\right)=P_{g}\left(t\right)-A\cdot D\\ D=|C\cdot P_{g}\left(t\right)-X\left(t\right)| \end{array} \end{array}$$

where ***A*** and ***C*** are the computed coefficient vectors. The bubble-net attacking operator imitates the prey attacking behavior of humpback whales, which consists of Shrinking encircling and Spiral updating processes. The Shrinking encircling process is similar to the previous stage, and each iteration of Spiral updating has a certain probability of indicating the simulated real situation with Equation 27:


(27) $$\begin{array}{l} X\left(t+1\right)=\{\begin{array}{cc} P_{g}\left(t\right)-A\cdot D & p<0.5\\ D^{{\;}^{\prime }}\cdot e^{bl}\cdot \cos\left(2\pi l\right)+P_{g}\left(t\right) & p\geq 0.5 \end{array} \end{array}$$

where ***D***′ is the distance between a robot and the current global maxima location, *b* is a constant for defining the shape of the logarithmic spiral, *p* and *l* are random numbers in the range of [0, 1] and [−1, 1], respectively. For IWOA, the upwind term ***V***^***u***^(***t***) is added to the position update. The update process being improved as Equation 28:


(28) $$\begin{array}{l} X\left(t+1\right)=D^{\prime }\cdot e^{bl}\cdot \cos\left(2\pi l\right)+X^{*}\left(t\right)+V^{u}(t) \end{array}$$

Searching for prey operator is the global search phase of the robot, and the t-moment algorithm randomly selects the robot position ***X***_*rand*_(*t*) as a reference for updating the position to maintain diversity. Compared with the standard PSO and IPSO, simulation results show that the success rate of the SWOA method for locating time-varying pollution sources is within the acceptable level only. However, the cost is lower, and the IWOA success rate is higher than the improved PSO (IPSO), but the localization efficiency is worse than IPSO.

Jiang et al. (2022) experimentally validated and compared the SWOA and IWOA algorithms with standard PSO and IPSO in a natural field. The experiments considered two typical time-varying sources (periodic and decaying sources) and two source locations (downwind zone and recirculation zone). The results show that IWOA has a significantly higher success rate and positioning accuracy in locating odor sources than IPSO. Zhou et al. (2022) made further experimental verification and compared the above algorithms. The results show that the IWOA method requires fewer average steps to locate the periodic source, has a higher success rate, and is more challenging to locate the decay source.

### Artificial ecosystem-based optimization

Artificial ecosystem-based optimization (AEO; Zhao et al., 2020) was inspired by the phenomenon of energy flowing along the food chain between producers, consumers, and decomposers in the ecosystem. This mechanism of maintaining a stable energy transfer for optimization algorithms has excellent potential, and experiments have shown that AEO outperforms its advanced counterparts in terms of optimization performance (Zhao et al., 2020). Especially for real-world engineering problems, AEO is more competitive than other reported methods in terms of convergence speed and calculation cost. In our previous work (Fu et al., 2021), we proposed an improved AEO algorithm through incorporating a discrete wind direction system to solve the uncertainty of concentration thresholds in different odor source environments during odor source localization. Producers, consumers, and decomposers share concentration and wind direction information throughout the process. The algorithm combines individual stochasticity and consumption factors to perform plume tracking strategies in low concentration regions, enhancing the algorithm's robustness.

In this algorithm, the producer is mainly employed for global search, and the *i*th robot position update equation in a population of *n* robots is as follows:


(29) $$\begin{array}{l} X_{i}\left(t+1\right)=\left(1-a\right)X_{n}\left(t\right)+aX_{rand}\left(t\right) \end{array}$$

where ***X***_*rand*_ is a random position in the search space and *a* is a linear weighting coefficient. There are three consumption strategies for consumers to become herbivores, carnivores, or omnivores, and the consumer. Robot will randomly choose to become one of them. Position change occurs after consumers “eat” different types of robots. The consumption process adds a consumption factor to avoid local optima. Subsequently, the decomposer will decompose the organisms to obtain concentration information, location information, and wind direction information to update the location of robot *i* as:


(30) $$\begin{array}{l} X_{i}\left(t+1\right)=X_{n}\left(t\right)+D\left[eX_{n}\left(t\right)-hX_{i}\left(t\right)\right]\\ \;+R_{d}\times W_{or}\times r_{4}\times \frac{-w_{i}}{\left|w_{ix}|+|w_{iy}\right|} \end{array}$$

where *D* is the decomposition factor, *e* and *h* are the weight coefficients, and *r*_4_ random coefficients in the range of [0, 1]. *R*_*d*_ is the decomposer wind direction factor, *w*_*i*_ is the wind speed vector at the current robot, *w*_*ix*_ and *w*_*iy*_ are the x-axis wind speed component vectors and y-axis wind speed component vectors at the current robot. *W*_*or*_ is the adaptive step size factor.

Finally, wind chasers are added and divided into two levels to perform different operations according to anemotaxis, with the following equation:


(31) $$\begin{array}{l} \{\begin{array}{c} X_{i}\left(t+1\right)=X_{i}\left(t\right)+R_{w}\times W_{or}\;\times r_{5}\times \frac{-w_{i}}{\left|w_{ix}|+|w_{iy}\right|},\;i=\left[1,\cdots \;,P_{w}\right]\\ P_{w}=C_{w}n \end{array} \end{array}$$

(32) $$\begin{array}{l} X_{i}\left(t+1\right)=X_{i}\left(t\right)+R_{w}\times W_{or}\;\times r_{5}\times \frac{-w_{i}}{\left|w_{ix}|+|w_{iy}\right|},\;r_{6}>Le \end{array}$$

where *R*_*w*_ is the wind direction factor of the wind chaser, *r*_5_ and *r*_6_ are uniformly distributed random numbers in the range of [0, 1], *P*_*w*_ is the number of robots that become chasers, *C*_*w*_ is the proportion chasers among all robots, and *Le* is the stratification factor. The simulation results show that the time spent and success rate of odor source locating has a greater advantage than CPSO and WU-PSO when the number of robots is small.

## Trends and challenges

### Other SI algorithms to be explored

In addition to the swarm intelligence and extension algorithms mentioned above, many widely discussed swarm intelligence algorithms have not been applied to odor source localization. The artificial fish swarm algorithm (AFS) is mainly based on fish foraging behavior in the natural environment. It adopts the bottom-up design idea, mainly using the three operators of artificial fish's foraging, clustering, and tailing finding, to construct the bottom behavior of individuals, which has the advantages of reliable global search ability and fast convergence (Tang et al., 2021). The bat algorithm is a random search algorithm that simulates the prey detection and obstacle avoidance behavior of bats in nature using echolocation. It can achieve single and multiple target search and localization in continuous space (Yang and Hossein Gandomi, 2012). The artificial bee colony optimization (ABC) algorithm simulates bees' foraging behavior. In addition to the primary selection mechanism of bees and simple interactions between bees, the ABC algorithm introduces local and global search mechanisms, making the algorithm capable of solving search problems in multi-objective fields (Karaboga and Basturk, 2007). Those algorithms have their own advantage to deal with different kinds of problems in various scenarios and are worthy to be considered for exploring in OSL applications according to No Free Lunch Theorem.

### From 2D to 3D

The study of OSL methods in three-dimensional space is more in line with the propagation characteristics of odors in the natural environment compared to that in two-dimensional plane. Therefore, it is more conducive to solving the needs of practical application. As far as equations are concerned, most swarm intelligence algorithms mentioned above can easily switch from 2D plane to 3D space. The difficulties may lie mainly in three aspects. (1) How to achieve the 3D spatial dynamic simulation of the sniffing robots. Robots in three-dimensional space can no longer be considered as mass points, and the large-scale flight operations of rotorcraft swarms will change the odor distribution on a macroscopic scale. How to simulate the change of odor distribution also needs to be taken into account. (2) How to solve the effect of UAV rotor blades on odor diffusion. The plume above the UAVs will be sucked by the rotor blades and emitted from below when UAVs passed through the smoke plume. In this case, the concentration, wind direction, and wind speed of the plume cannot be accurately measured. How to install the sensor at the right position to measure the accurate value will be the key consideration of scholars. (3) How to avoid mutual collision of UAVs in 3D turbulent space. Swarms of drones flying in dynamic turbulence will be susceptible to position shifts and collisions, so more conservative strategies to avoid mutual collisions need to be considered. To solve these problems, some researchers have turned to study airflow in 3D environments to extend the chemical sensing capabilities of mobile robots to 3D environments (Ishida et al., 1999; Feng et al., 2018). The rapid development of UAV technology has provided an excellent platform, which has led to the 3D spatial OSL problem based on the UAV platform receiving the attention of most researchers (Jing et al., 2021). Preliminary research results have been achieved (Neumann et al., 2013; Pobkrut et al., 2016; Villa et al., 2016; Luo et al., 2018; Gunawardena et al., 2021; Liu et al., 2022). However, compared with OSL in two dimensions, these results are still far from adequate and need further exploration.

### Fusion of multimodal sensing information

Biological evidence proves that other sensing modalities also play an essential role in odor source localization of organisms. Sensing inputs of odor concentration information and wind information are now widely used for odor source localization. Nevertheless, odor sensors have certain information blindness. The sensor accuracy sometimes cannot meet the operational requirements, and the slow response and prolonged recovery time of sensors limit the localization efficiency. On the other hand, wind direction information cannot independently apply to odor source localization. When the chemical concentration sensor is not working, the fusion of information from multiple sensing modules ensures the iterative process. This will enhance the search performance and accuracy of the system. There have been few results in visual research (Jing et al., 2021), and Ishida et al. (2006) proposed a new method for locating odor sources using a combination of visual, odor, and wind direction information. The method assembles a mobile robot with CMOS cameras, odor sensors, and airflow sensors. It employs a behavior-based navigation strategy to successfully locate a bottle containing the source of a leaking odor in an indoor experiment. Shen et al. (2021) designed a multi-sensor information fusion decision strategy and incorporated the vision and olfactory sensor fusion for tracking plumes and declaring the odor source in a partial 3D diffusion environment. In addition to visual sensors, we hope that sensors related to temperature gradient, wind speed and hearing as well as other complex sensors [IMU, Insect-behavior-based Olfactory Sensor (Horibe et al., 2021), Lidar] can be further investigated in the future for applications in certain special situations, such as fire scenes, narrow caves, etc. Corresponding to multimodal sensing information fusion is a multi-source, asynchronous decision framework. How to develop an optimal strategy based on this requires in-depth study.

## Conclusion

Swarm intelligence algorithms solve complex optimization problems through the communication and collaboration of multiple simple individuals, emerging as a distributed, self-organizing group intelligence. Multi-robot odor source localization is for searching the optimal odor concentration in the target space by multiple robots. It is coherent in purpose with the swarm intelligence optimization algorithm. Therefore, employing swarm intelligence optimization algorithms to solve OSL problems has become mainstream in the last two decades. This review briefly reviews the history and basic concepts of OSL research and summarizes the core issues and trends in OSL field. Multi-robotics for localizing multiple odor sources in natural environments using chemotaxis-anemotaxis of multi-sensing information is the leading research direction. To achieve this goal, scholars have introduced standard PSO and its various variant versions into the research field, including applicability improvements to standard PSO and hybrid algorithms that combine other optimization algorithms. Further, various nature-inspired SI algorithms have also been introduced into OSL, such as CSA, ACO, GWO, GSO, WOA, and AEO. SI-based multi-robots for odor source localization is on the rise. Finally, we believe that based on the principle of no free lunch theorem, other swarm intelligence algorithms, through adaptive modifications, also have the potential to be introduced into this research area to solve specific problems.

## Author contributions

JW and JF: conceptualization and writing—review and editing. JW, YL, RL, and JF: methodology and investigation. JW, YL, and RL: writing—original draft preparation. JW: visualization. JF: supervision and project administration. All authors have read and agreed to the published version of the manuscript.

## Funding

This research was supported by the National Natural Science Foundation of China, Grant No. 61305030 and Xinmiao Talents Program of Zhejiang Province, Grant No. 2021R408028.

## Conflict of interest

The authors declare that the research was conducted in the absence of any commercial or financial relationships that could be construed as a potential conflict of interest.

## Publisher's note

All claims expressed in this article are solely those of the authors and do not necessarily represent those of their affiliated organizations, or those of the publisher, the editors and the reviewers. Any product that may be evaluated in this article, or claim that may be made by its manufacturer, is not guaranteed or endorsed by the publisher.

## References

[B1] Balkovsky E. Shraiman B. (2002). Olfactory search at high Reynolds number. Proc. Natl. Acad. Sci. U. S. A. 99, 12589–12593. 10.1073/pnas.192393499 12228727 PMC130504

[B2] Cao M.-L. Meng Q.-H. Wang X.-W. Luo B. Zeng M. Li W. (2013). “Localization of multiple odor sources *via* selective olfaction and adapted ant colony optimization algorithm,” in Proceedings of the 2013 IEEE International Conference on Robotics and Biomimetics (ROBIO) (Shenzhen: IEEE), 1222–1227. 10.1109/ROBIO.2013.6739631

[B3] Charlton R. E. Cardé R. T. (1990). Orientation of male gypsy moths, *Lymantria Dispar* (L.), to pheromone sources: the role of olfactory and visual cues. J. Insect Behav. 3, 443–469. 10.1007/BF01052011

[B4] Che H. Shi C. Xu X. Li J. Wu B. (2018). “Research on improved ACO algorithm-based multi-robot odor source localization,” in Proceedings of the 2018 2nd International Conference on Robotics and Automation Sciences (ICRAS) (Wuhan: IEEE), 1–5. 10.1109/ICRAS.2018.8443237

[B5] Chen X. Huang J. (2019). Odor source localization algorithms on mobile robots: a review and future outlook. Robot. Auton. Syst. 112, 123–136. 10.1016/j.robot.2018.11.014

[B6] Chen Y. Cai H. Chen Z. Feng Q. (2017). Using multi-robot active olfaction method to locate time-varying contaminant source in indoor environment. Build. Environ. 118, 101–112. 10.1016/j.buildenv.2017.03.03032288120

[B7] Dorigo M. Maniezzo V. Colorni A. (1996). Ant system: optimization by a colony of cooperating agents. IEEE Trans. Syst. Man Cybern. B Cybern. 26, 29–41. 10.1109/3477.484436 18263004

[B8] Fan H. Arain M. A. Bennett V. H. Schaffernicht E. Lilienthal A. J. (2017). “Improving gas dispersal simulation for mobile robot olfaction: using robot-created occupancy maps and remote gas sensors in the simulation loop,” in Proceedings of the 2017 ISOCS/IEEE International Symposium on Olfaction and Electronic Nose (ISOEN) (Montreal: IEEE), 1–3. 10.1109/ISOEN.2017.7968874

[B9] Farrell J. A. Murlis J. Long X. Li W. Carde R. T. (2002). Filament-based atmospheric dispersion model to achieve short time-scale structure of odor plumes. Environ. FLUID Mech. 2, 143–169. 10.1023/A:1016283702837

[B10] Feng Q. Cai H. Chen Z. Yang Y. Lu J. Li F.. (2019a). Experimental study on a comprehensive particle swarm optimization method for locating contaminant sources in dynamic indoor environments with mechanical ventilation. ENERGY Build. 196, 145–156. 10.1016/j.enbuild.2019.03.032 32288120 PMC7111221

[B11] Feng Q. Cai H. Li F. Liu X. Liu S. Xu J. (2019b). An improved particle swarm optimization method for locating time-varying indoor particle sources. Build. Environ. 147, 146–157. 10.1016/j.buildenv.2018.10.008 32287987 PMC7117037

[B12] Feng Q. Cai H. Li F. Yang Y. Chen Z. (2018). Locating time-varying contaminant sources in 3D indoor environments with three typical ventilation systems using a multi-robot active olfaction method. Build. Simul. 11, 597–611. 10.1007/s12273-017-0424-6

[B13] Feng Q. Cai H. Yang Y. Xu J. Jiang M. Li F.. (2020). An experimental and numerical study on a multi-robot source localization method independent of airflow information in dynamic indoor environments. Sustain. Cities Soc. 53, 101897. 10.1016/j.scs.2019.101897

[B14] Feng Q. Zhang C. Lu J. Cai H. Chen Z. Yang Y.. (2019c). Source localization in dynamic indoor environments with natural ventilation: an experimental study of a particle swarm optimization-based multi-robot olfaction method. Build. Environ. 161, 106228. 10.1016/j.buildenv.2019.106228

[B15] Ferri G. Caselli E. Mattoli V. Mondini A. Mazzolai B. Dario P. (2007). “Explorative particle swarm optimization method for gas/odor source localization in an indoor environment with no strong airflow,” in Proceedings of the 2007 Ieee International Conference on Robotics and Biomimetics, Vols 1-5 (New York, NY: IEEE), 841–846. 10.1109/ROBIO.2007.4522272

[B16] Francis A. Li S. Griffiths C. Sienz J. (2022). Gas source localization and mapping with mobile robots: a review. J. Field Robot. 2022, 22109. 10.1002/rob.22109

[B17] Fu J. Shen L. Liu R. (2021). An indoor odor source locating method for multi-robot active olfaction based on improved AEO. Chin. J. Sens. Actuators 34, 1406–1411. 10.3969/j.issn.1004-1699.2021.10.020

[B18] Fu Z. Chen Y. Ding Y. He D. (2019). Pollution source localization based on multi-UAV cooperative communication. IEEE Access 7, 29304–29312. 10.1109/ACCESS.2019.2900475

[B19] Gaurav K. Kumar A. Singh R. (2020). Single and multiple odor source localization using hybrid nature-inspired algorithm. Sādhanā 45, 83. 10.1007/s12046-020-1318-3

[B20] Genovese V. Dario P. Magni R. Odetti L. (1992). “Self organizing behavior and swarm intelligence in a pack of mobile miniature robots in search of pollutants,” in Proceedings of the IEEE/RSJ International Conference on Intelligent Robots and Systems (Raleigh: IEEE), 1575–1582. 10.1109/IROS.1992.594225

[B21] Ghalia M. B. (2008). “Particle swarm optimization with an improved exploration-exploitation balance,” in 2008 51st Midwest Symposium on Circuits and Systems (Knoxville, TN: IEEE), 759–762. 10.1109/MWSCAS.2008.4616910

[B22] Gong D.-W. Zhang Y. Qi C.-L. (2012). Localising odour source using multi-robot and anemotaxis-based particle swarm optimisation. IET Control Theory Appl. 6, 1661–1670. 10.1049/iet-cta.2011.0513

[B23] Gunawardena N. Leang K. K. Pardyjak E. (2021). Particle swarm optimization for source localization in realistic complex urban environments. Atmos. Environ. 262, 118636. 10.1016/j.atmosenv.2021.118636

[B24] Hayes A. T. Martinoli A. Goodman R. M. (2002). Distributed odor source localization. IEEE Sens. J. 2, 260–271. 10.1109/JSEN.2002.800682

[B25] Hölldobler B. Hölldobler R. Wilson E. O. Wilson H. C. (1990). The Ants. Cambridge, MA: Harvard University Press. 10.1007/978-3-662-10306-7

[B26] Horibe J. Ando N. Kanzaki R. (2021). Odor-searching Robot with Insect-behavior-based Olfactory Sensor. Sens. Mater. 33, 4185. 10.18494/SAM.2021.3369

[B27] Ishida H. Kobayashi A. Nakamoto T. Moriizumi T. (1999). Three-dimensional odor compass. IEEE Trans. Robot. Autom. 15, 251–257. 10.1109/70.760346

[B28] Ishida H. Nakayama G. Nakamoto T. Moriizumi T. (2005). Controlling a gas/odor plume-tracking robot based on transient responses of gas sensors. IEEE Sens. J. 5, 537–545. 10.1109/JSEN.2004.839597

[B29] Ishida H. Suetsugu K. Nakamoto T. Moriizumi T. (1994). Study of autonomous mobile sensing system for localization of odor source using gas sensors and anemometric sensors. Sens. Actuators Phys. 45, 153–157. 10.1016/0924-4247(94)00829-9

[B30] Ishida H. Tanaka H. Taniguchi H. Moriizumi T. (2006). Mobile robot navigation using vision and olfaction to search for a gas/odor source. Auton. Robots 20, 231–238. 10.1007/s10514-006-7100-5

[B31] Ishida H. Wada Y. Matsukura H. (2012). Chemical sensing in robotic applications: a review. IEEE Sens. J. 12, 3163–3173. 10.1109/JSEN.2012.2208740

[B32] Jain U. Tiwari R. Godfrey W. W. (2018). “Odor source localization by concatenating particle swarm optimization and grey wolf optimizer,” in Proceedings of the Advanced Computational and Communication Paradigms (Singapore: Springer), 145–153. 10.1007/978-981-10-8237-5_14

[B33] Jain U. Tiwari R. Godfrey W. W. (2019). Multiple odor source localization using diverse-PSO and group-based strategies in an unknown environment. J. Comput. Sci. 34, 33–47. 10.1016/j.jocs.2019.04.008

[B34] Jatmiko W. Jovan F. Dhiemas R. Y. S. Alvissalim M. S. Febrian A. Widiyanto D.. (2016). PSO algorithm for single and multiple odor sources localization problems: progress and challenge. Int. J. Smart Sens. Intell. Syst. 9, 1431–1478. 10.21307/ijssis-2017-925

[B35] Jatmiko W. Nugraha A. Efendi R. Pambuko W. Mardian R. Sekiyama K.. (2009a). Localizing multiple odor sources in a dynamic environment based on modified niche particle swarm optimization with flow of wind. WSEAS Trans. Syst. 8, 1187–1196. Available online at: http://www.wseas.us/e-library/transactions/systems/2009/32-562.pdf

[B36] Jatmiko W. Pambuko W. Mursanto P. Muis A. Kusumoputro B. Sekiyama K.. (2009b). “Localizing multiple odor sources in dynamic environment using ranged subgroup PSO with flow of wind based on open dynamic engine library,” in Proceedings of the 2009 International Symposium on Micro-NanoMechatronics and Human Science (Nagoya: IEEE), 602–607. 10.1109/MHS.2009.5351761

[B37] Jatmiko W. Sekiyama K. Fukuda T. (2006a). “A mobile robots PSO-based for odor source localization in extreme dynamic advection-diffusion environment with obstacle,” in Proceedings of the 2006 IEEE SENSORS (Daegu: IEEE), 526–529. 10.1109/IROS.2006.282092

[B38] Jatmiko W. Sekiyama K. Fukuda T. (2006b). “A particle swarm-based mobile sensor network for odor source localization in a dynamic environment,” in Proceedings of the Distributed Autonomous Robotic Systems 7 (Tokyo: Springer Japan), 71–80. 10.1007/4-431-35881-1_8

[B39] Jatmiko W. Sekiyama K. Fukuda T. (2007). A PSO-based mobile robot for odor source localization in dynamic advection-diffusion with obstacles environment: theory, simulation and measurement. IEEE Comput. Intell. Mag. 2, 37–51. 10.1109/MCI.2007.353419

[B40] Jiang M. Liao Y. Guo X. Cai H. Jiang W. Yang Z.. (2022). A comparative experimental study of two multi-robot olfaction methods: towards locating time-varying indoor pollutant sources. Build. Environ. 207, 108560. 10.1016/j.buildenv.2021.108560

[B41] Jing T. Meng Q. Ishida H. (2021). Recent progress and trend of robot odor source localization. IEEJ Trans. Electr. Electron. Eng. 16, 938–953. 10.1002/tee.23364

[B42] Karaboga D. Basturk B. (2007). A powerful and efficient algorithm for numerical function optimization: artificial bee colony (ABC) algorithm. J. Glob. Optim. 39, 459–471. 10.1007/s10898-007-9149-x

[B43] Kennedy J. Eberhart R. (1995). “Particle swarm optimization,” in Proceedings of the ICNN'95 - International Conference on Neural Networks, vol.4 (Perth: IEEE), 1942–1948.

[B44] Kirkpatrick S. Gelatt C. D. Vecchi M. P. (1983). Optimization by simulated annealing. Science 220, 671–680. 10.1126/science.220.4598.671 17813860

[B45] Kowadlo G. Russell R. A. (2008). Robot odor localization: a taxonomy and survey. Int. J. Robot. Res. 27, 869–894. 10.1177/0278364908095118

[B46] Krishnanand K. N. Amruth P. Guruprasad M. H. Bidargaddi S. V. Ghose D. (2006). “Glowworm-inspired robot swarm for simultaneous taxis towards multiple radiation sources,” in Proceedings of the 2006 Ieee International Conference on Robotics and Automation (icra), Vols 1-10 (New York, NY: IEEE), 958–963. 10.1109/ROBOT.2006.1641833

[B47] Krishnanand K. N. Ghose D. (2005). “Detection of multiple source locations using a glowworm metaphor with applications to collective robotics,” in Proceedings of the 2005 IEEE Swarm Intelligence Symposium, 2005. SIS 2005. (Pasadena: IEEE), 84–91. 10.1109/SIS.2005.1501606

[B48] Krishnanand K. N. Ghose D. (2009). Glowworm swarm optimization for simultaneous capture of multiple local optima of multimodal functions. Swarm Intell. 3, 87–124. 10.1007/s11721-008-0021-5

[B49] Kwa H. L. Leong Kit J. Bouffanais R. (2022). Balancing collective exploration and exploitation in multi-agent and multi-robot systems: a review. Front. Robot. AI 8, 771520. 10.3389/frobt.2021.771520 35178430 PMC8844516

[B50] Li F. Meng Q.-H. Bai S. Li J.-G. Popescu D. (2008). “Probability-PSO algorithm for multi-robot based odor source localization in ventilated indoor environments,” in Proceedings of the Intelligent Robotics and Applications (Berlin; Heidelberg: Springer), 1206–1215. 10.1007/978-3-540-88513-9_128

[B51] Li F. Meng Q.-H. Li J.-G. Zeng M. (2010). P-PSO algorithm based multi-robot odor source search in ventilated indoor environment with obstacles: P-PSO algorithm based multi-robot odor source search in ventilated indoor environment with obstacles. Acta Autom. Sin. 35, 1573–1579. 10.3724/SP.J.1004.2009.01573

[B52] Li J.-G. Yang J. Zhou J.-Y. Liu J. Lu G.-D. (2015). Mapping odour sources with a mobile robot in a time variant airflow environment. Austrian Contrib. Vet. Epidemiol. 8, 7. 10.5281/zenodo.33825

[B53] Li W. Farrell J. Pang S. Arrieta R. (2006). Moth-inspired chemical plume tracing on an autonomous underwater vehicle. IEEE Trans. Robot. 22, 292–307. 10.1109/TRO.2006.870627

[B54] Lilienthal A. J. Loutfi A. Duckett T. (2006). Airborne chemical sensing with mobile robots. SENSORS 6, 1616–1678. 10.3390/s611161630736489

[B55] Liu Y. Zhao X. Xu J. Zhu S. Su D. (2022). Rapid location technology of odor sources by multi-UAV. J. Field Robot. 39, 600–616. 10.1002/rob.22066

[B56] Lu Q. Han Q.-L. (2010). “A distributed coordination control scheme for odor source localization,” in Proceedings of the IECON 2010 - 36th Annual Conference on IEEE Industrial Electronics Society (Glendale: IEEE), 1413–1418. 10.1109/IECON.2010.5675475

[B57] Lu Q. Han Q.-L. Liu S. (2014a). A finite-time particle swarm optimization algorithm for odor source localization. Inf. Sci. 277, 111–140. 10.1016/j.ins.2014.02.01023033435

[B58] Lu Q. Han Q.-L. Liu S. (2016). A cooperative control framework for a collective decision on movement behaviors of particles. IEEE Trans. Evol. Comput. 20, 859–873. 10.1109/TEVC.2016.2526656

[B59] Lu Q. Han Q.-L. Xie X. Liu S. (2014b). A finite-time motion control strategy for odor source localization. IEEE Trans. Ind. Electron. 61, 5419–5430. 10.1109/TIE.2014.2301751

[B60] Lu Q. Liu S. Xie X. Wang J. (2013). Decision making and finite-time motion control for a group of robots. IEEE Trans. Cybern. 43, 738–750. 10.1109/TSMCB.2012.2215318 23033435

[B61] Luo B. Meng Q.-H. Wang J.-Y. Zeng M. (2018). A flying odor compass to autonomously locate the gas source. IEEE Trans. Instrum. Meas. 67, 137–149. 10.1109/TIM.2017.2759378

[B62] Luo D. Zou Y. Zhuang J. (2008). Multi-robot odor source localization strategy based on a modified ant colony algorithm. Robot 30, 536–541. Available online at: https://robot.sia.cn/EN/Y2008/V30/I6/536

[B63] Mamduh S. M. Kamarudin K. Shakaff A. Y. M. Zakaria A. Visvanathan R. Yeon A. S. A.. (2018). Gas source localization using grey wolf optimizer. J. Telecommun. Electron. Comput. Eng. 10, 95–98. Available online at: https://jtec.utem.edu.my/jtec/article/view/4130/2956

[B64] Marques L. Nunes U. de Almeida A. T. (2006). Particle swarm-based olfactory guided search. Auton. Robots 20, 277–287. 10.1007/s10514-006-7567-0

[B65] Meng Q.-H. Li J.-C. Li F. Zeng M. (2006). “Mobile robots odor localization with an improved ant colony algorithm,” in Proceedings of the 2006 IEEE International Conference on Robotics and Biomimetics (Kunming: IEEE), 959–964. 10.1109/ROBIO.2006.340358

[B66] Meng Q.-H. Yang W.-X. Wang Y. Zeng M. (2010). “Multi-robot odor-plume tracing in indoor natural airflow environments using an improved ACO algorithm,” in Proceedings of the 2010 IEEE International Conference on Robotics and Biomimetics (Tianjin: IEEE), 110–115. 10.1109/ROBIO.2010.5723312

[B67] Meng Q.-H. Yang W.-X. Wang Y. Zeng M. (2011). Collective odor source estimation and search in time-variant airflow environments using mobile robots. Sensors 11, 10415–10443. 10.3390/s111110415 22346650 PMC3274292

[B68] Mirjalili S. Lewis A. (2016). The whale optimization algorithm. Adv. Eng. Softw. 95, 51–67. 10.1016/j.advengsoft.2016.01.008

[B69] Mirjalili S. Mirjalili S. M. Lewis A. (2014). Grey wolf optimizer. Adv. Eng. Softw. 69, 46–61. 10.1016/j.advengsoft.2013.12.007

[B70] Murlis J. Elkinton J. S. Cardé R. T. (1992). Odor plumes and how insects use them. Annu. Rev. Entomol. 37, 505–532. 10.1146/annurev.en.37.010192.002445

[B71] Neumann P. P. Bennetts V. H. Lilienthal A. J. Bartholmai M. Schiller J. H. (2013). Gas source localization with a micro-drone using bio-inspired and particle filter-based algorithms. Adv. Robot. 27, 725–738. 10.1080/01691864.2013.779052

[B72] Pobkrut T. Eamsa-ard T. Kerdcharoen T. (2016). “Sensor drone for aerial odor mapping for agriculture and security services,” in Proceedings of the 2016 13th International Conference on Electrical Engineering/Electronics, Computer, Telecommunications and Information Technology (ECTI-CON) (Chiang Mai: IEEE), 1–5. 10.1109/ECTICon.2016.7561340

[B73] Russell R. A. Bab-Hadiashar A. Shepherd R. L. Wallace G. G. (2003). A comparison of reactive robot chemotaxis algorithms. Robot. Auton. Syst. 45, 83–97. 10.1016/S0921-8890(03)00120-9

[B74] Shen X. Yuan J. Shan Y. (2021). A novel plume tracking method in partial 3D diffusive environments using multi-sensor fusion. Expert Syst. Appl. 178, 114993. 10.1016/j.eswa.2021.114993

[B75] Sinha A. Kumar R. Kaur R. Mishra R. K. (2020). Consensus-based odor source localization by multiagent systems under resource constraints. IEEE Trans. Cybern. 50, 3254–3263. 10.1109/TCYB.2019.2924328 31331900

[B76] Smyth W. Moum J. (2001). 3D turbulence. Encycl. Ocean Sci. Acad. Press 6, 2947–2955. 10.1006/rwos.2001.0134

[B77] Tang J. Liu G. Pan Q. (2021). A review on representative swarm intelligence algorithms for solving optimization problems: applications and trends. IEEECAA J. Autom. Sin. 8, 1627–1643. 10.1109/JAS.2021.1004129

[B78] Van den Bergh F. Engelbrecht A. P. (2002). “A new locally convergent particle swarm optimiser,” in Proceedings of the IEEE International Conference on System Man and Cybernetics (Piscataway, NJ: IEEE), 6. 10.1109/ICSMC.2002.1176018

[B79] Villa T. F. Salimi F. Morton K. Morawska L. Gonzalez F. (2016). Development and validation of a UAV based system for air pollution measurements. Sensors 16, 2202. 10.3390/s16122202 28009820 PMC5191180

[B80] Wang J. Zhang R. Yan Y. Dong X. Li J. M. (2017). Locating hazardous gas leaks in the atmosphere *via* modified genetic, MCMC and particle swarm optimization algorithms. Atmos. Environ. 157, 27–37. 10.1016/j.atmosenv.2017.03.009

[B81] Wang L. Pang S. (2021). Robotic odor source localization *via* adaptive bio-inspired navigation using fuzzy inference methods. Robot. Auton. Syst. 2021, 103914. 10.1016/j.robot.2021.103914

[B82] Wang W. Cao M. Ma S. Ren C. Zhu X. Lu H. (2016). “Multi-robot odor source search based on Cuckoo search algorithm in ventilated indoor environment,” in Proceedings of the 2016 12th World Congress on Intelligent Control and Automation (WCICA) (Guilin: IEEE), 1496–1501. 10.1109/WCICA.2016.7578817

[B83] Wu Y. Wang Z. (2021). “An improved Cuckoo search algorithm for multiple odor sources localization,” in Proceedings of the 13th International Conference on Agents and Artificial Intelligence - Vol 2 (Setubal: Scitepress), 708–715. 10.5220/0010231707080715

[B84] Xie X.-F. Zhang W.-J. Yang Z.-L. (2002). “Dissipative particle swarm optimization,” in Proceedings of the 2002 Congress on Evolutionary Computation. CEC'02 (Cat. No.02TH8600) (Honolulu, HI: IEEE), 1456–1461.

[B85] Yan Y. Zhang R. Wang J. Li J. (2018). Modified PSO algorithms with “Request and Reset” for leak source localization using multiple robots. Neurocomputing 292, 82–90. 10.1016/j.neucom.2018.02.078

[B86] Yang X. Hossein Gandomi A. (2012). Bat algorithm: a novel approach for global engineering optimization. Eng. Comput. 29, 464–483. 10.1108/02644401211235834

[B87] Yang X.-S. Deb S. (2009). “Cuckoo search *via* lévy flights,” in Proceedings of the 2009 World Congress on Nature Biologically Inspired Computing (NaBIC) (Coimbatore: IEEE), 210–214. 10.1109/NABIC.2009.5393690

[B88] Yang Y. Zhang B. Feng Q. Cai H. Jiang M. Zhou K.. (2019). Towards locating time-varying indoor particle sources: development of two multi-robot olfaction methods based on whale optimization algorithm. Build. Environ. 166, 106413. 10.1016/j.buildenv.2019.106413

[B89] Yee E. Chan R. Kosteniuk P. R. Chandler G. M. Biltoft C. A. Bowers J. F. (1994). Experimental measurements of concentration fluctuations and scales in a dispersing plume in the atmospheric surface layer obtained using a very fast response concentration detector. J. Appl. Meteorol. Climatol. 33, 996–1016. 10.1175/1520-0450(1994)033<0996:EMOCFA>2.0.CO;2

[B90] Zainal N. Zain A. M. Radzi N. H. M. Udin A. (2013). Glowworm swarm optimization (GSO) algorithm for optimization problems: a state-of-the-art review. Appl. Mech. Mater. 421, 507–511. 10.4028/www.scientific.net/AMM.421.507

[B91] Zhang J. Gong D. Zhang Y. (2014). A niching PSO-based multi-robot cooperation method for localizing odor sources. Neurocomputing 123, 308–317. 10.1016/j.neucom.2013.07.025

[B92] Zhang Y. Gong D. Hu Y. Zhang J. (2014). A PSO-based multi-robot search method for odor source in indoor environment with noise. Acta Electonica Sin. 42, 70. 10.3969/j.issn.0372-2112.2014.01.011

[B93] Zhang Y. Ma X. Miao Y. (2011). “Localization of multiple odor sources using modified glowworm swarm optimization with collective robots,” in Proceedings of the 30th Chinese Control Conference (Yantai: IEEE), 1899–1904. 10.1109/CCDC.2011.5968545

[B94] Zhang Y. Ma X. Miao Y. (2015a). Multiple chemical sources localization using virtual physics-based robots with release strategy. Math. Probl. Eng. 2015, 1–16. 10.1155/2015/678451

[B95] Zhang Y. Zhang J. Hao G. Zhang W. (2015b). “Localizing odor source with multi-robot based on hybrid particle swarm optimization,” in Proceedings of the 2015 11th International Conference on Natural Computation (ICNC) (Zhangjiajie: IEEE), 902–906. 10.1109/ICNC.2015.7378110

[B96] Zhao W. Wang L. Zhang Z. (2020). Artificial ecosystem-based optimization: a novel nature-inspired meta-heuristic algorithm. Neural Comput. Appl. 32, 9383–9425. 10.1007/s00521-019-04452-x

[B97] Zhou S. Zhang C. Cai H. Zhang B. Feng Q. Feng L.. (2022). Locating a time-varying contaminant source in naturally ventilated indoor environments: an experimental study to find effective multi-robot olfaction methods. Build. Environ. 216, 108954. 10.1016/j.buildenv.2022.108954

[B98] Zou Y. Luo D. (2008). “A modified ant colony algorithm used for multi-robot odor source localization,” in Proceedings of the Advanced Intelligent Computing Theories and Applications. With Aspects of Artificial Intelligence (Berlin, Heidelberg: Springer), 502–509. 10.1007/978-3-540-85984-0_60

[B99] Zou Y. Luo D. Chen W. (2009). “Swarm robotic odor source localization using ant colony algorithm,” in Proceedings of the 2009 IEEE International Conference on Control and Automation (Christchurch: IEEE), 792–796. 10.1109/ICCA.2009.5410516

